# The Vitamin C, Thiamine and Steroids in Sepsis (VICTAS) Protocol: a prospective, multi-center, double-blind, adaptive sample size, randomized, placebo-controlled, clinical trial

**DOI:** 10.1186/s13063-019-3254-2

**Published:** 2019-04-05

**Authors:** David N. Hager, Michael H. Hooper, Gordon R. Bernard, Laurence W. Busse, E. Wesley Ely, Alpha A. Fowler, David F. Gaieski, Alex Hall, Jeremiah S. Hinson, James C. Jackson, Gabor D. Kelen, Mark Levine, Christopher J. Lindsell, Richard E. Malone, Anna McGlothlin, Richard E. Rothman, Kert Viele, David W. Wright, Jonathan E. Sevransky, Greg S. Martin

**Affiliations:** 1 Division of Pulmonary & Critical Care Medicine, Department of Medicine, Johns Hopkins Hospital, Johns Hopkins University, 1800 Orleans Street, Suite 9121, Baltimore, MD 21287 USA; 2 Division of Pulmonary & Critical Care Medicine, Department of Medicine, Eastern Virginia Medical School and Sentara Healthcare, Norfolk, VA USA; 3 Division of Pulmonary & Critical Care Medicine, Department of Medicine, Vanderbilt University School of Medicine, Nashville, TN USA; 4 Division of Pulmonary, Allergy, Critical Care, and Sleep Medicine, Department of Medicine, Emory University, Atlanta, GA USA; 5 Division of Pulmonary & Critical Care, Department of Medicine, Vanderbilt University Medical Center, Nashville, TN USA; 6 Critical Illness, Brain Dysfunction, and Survivorship (CIBS) Center, Vanderbilt University Medical Center, Nashville, TN USA; 7 Tennessee Valley Veteran’s Affairs Geriatric Research Education Clinical Center (GRECC), Nashville, TN USA; 8 Division of Pulmonary Disease & Critical Care Medicine, Department of Internal Medicine, The VCU Johnson Center for Critical Care and Pulmonary Research, Virginia Commonwealth University School of Medicine, Richmond, VA USA; 9 Department of Emergency Medicine, Sidney Kimmel Medical College, Thomas Jefferson University, Philadelphia, PA USA; 10 Department of Emergency Medicine, Emory University, Atlanta, GA USA; 11 Grady Memorial Hospital, Atlanta, GA USA; 12 Department of Emergency Medicine, Johns Hopkins University, Baltimore, MD USA; 13 Department of Psychiatry, Vanderbilt University School of Medicine, Nashville, TN USA; 14 Molecular & Clinical Nutrition Section, Intramural Research Program, National Institute of Diabetes and Digestive and Kidney Diseases, National Institutes of Health, 10 Center Drive, Bethesda, MD USA; 15 Department of Biostatistics, Vanderbilt University Medical Center, Nashville, TN USA; 16 Investigational Drug Service, Vanderbilt University Medical Center, Nashville, TN USA; 17 Berry Consultants, LLC, Austin, TX USA

**Keywords:** Vitamin C, Thiamine, Hydrocortisone, Sepsis, Septic shock, Mortality, Randomized controlled trial

## Abstract

**Background:**

Sepsis accounts for 30% to 50% of all in-hospital deaths in the United States. Other than antibiotics and source control, management strategies are largely supportive with fluid resuscitation and respiratory, renal, and circulatory support. Intravenous vitamin C in conjunction with thiamine and hydrocortisone has recently been suggested to improve outcomes in patients with sepsis in a single-center before-and-after study. However, before this therapeutic strategy is adopted, a rigorous assessment of its efficacy is needed.

**Methods:**

The Vitamin C, Thiamine and Steroids in Sepsis (VICTAS) trial is a prospective, multi-center, double-blind, adaptive sample size, randomized, placebo-controlled trial. It will enroll patients with sepsis causing respiratory or circulatory compromise or both. Patients will be randomly assigned (1:1) to receive intravenous vitamin C (1.5 g), thiamine (100 mg), and hydrocortisone (50 mg) every 6 h or matching placebos until a total of 16 administrations have been completed or intensive care unit discharge occurs (whichever is first). Patients randomly assigned to the comparator group are permitted to receive open-label stress-dose steroids at the discretion of the treating clinical team. The primary outcome is consecutive days free of ventilator and vasopressor support (VVFDs) in the 30 days following randomization. The key secondary outcome is mortality at 30 days. Sample size will be determined adaptively by using interim analyses with pre-stated stopping rules to allow the early recognition of a large mortality benefit if one exists and to refocus on the more sensitive outcome of VVFDs if an early large mortality benefit is not observed.

**Discussion:**

VICTAS is a large, multi-center, double-blind, adaptive sample size, randomized, placebo-controlled trial that will test the efficacy of vitamin C, thiamine, and hydrocortisone as a combined therapy in patients with respiratory or circulatory dysfunction (or both) resulting from sepsis. Because the components of this therapy are inexpensive and readily available and have very favorable risk profiles, demonstrated efficacy would have immediate implications for the management of sepsis worldwide.

**Trial registration:**

ClinicalTrials.gov Identifier: NCT03509350.

First registered on April 26, 2018, and last verified on December 20, 2018.

Protocol version: 1.4, January 9, 2019

**Electronic supplementary material:**

The online version of this article (10.1186/s13063-019-3254-2) contains supplementary material, which is available to authorized users.

## Background

Sepsis is an inflammatory syndrome with life-threatening organ dysfunction resulting from a dysregulated host response to infection [1]. Each year in the United States, where the incidence is increasing, there are an estimated 1,750,000 cases, half of which require intensive care unit (ICU) admission [2–4]. These cases account for 30% to 50% of all in-hospital deaths, making sepsis the third leading cause of death in the United States and the most expensive reason for hospitalization; annual in-patient expenditure is nearly $24 billion [5–7]. Those who survive endure significant reductions in physical, emotional, and cognitive quality of life [8, 9].

Current management strategies for patients with sepsis include early aggressive fluid resuscitation, early appropriate antibiotics, hemodynamic support with vasopressors, and the identification and control of infected sites [10, 11]. Although outcomes have improved with the bundled deployment of these strategies [12–15], mortality remains high at 20–30% [4, 16]. Cost-effective and low-risk therapeutic approaches to reduce the morbidity and mortality of sepsis are needed. For this reason, the 32% absolute mortality reduction observed in a recent study of a combination therapy, including intravenous vitamin C (1.5 g every 6 h), thiamine (200 mg every 12 h), and hydrocortisone (50 mg every 6 h), has attracted significant attention and enthusiasm from the lay press, patient advocacy groups, private foundations, and some clinicians [17–20]. By contrast, the sobering experiences of more than 100 phase 2 and 3 clinical trials of promising pharmacological agents, none of which demonstrated reproducible benefits among patients with sepsis, have caused others to have a more reserved response [21–23]. Many providers will await a more rigorous assessment of this three-drug regimen before adopting its use [24–26].

Although well-designed, randomized controlled trials of the vitamin C, thiamine, and hydrocortisone regimen have not been completed, the reported beneficial effects are biologically plausible. Vitamin C is an essential micronutrient not synthesized by humans [27], is an enzymatic cofactor in the endogenous synthesis of norepinephrine, and is a well-known antioxidant. Indeed, oxidative stress is part of the sepsis syndrome where an overproduction of reactive oxygen species causes lipid peroxidation, endothelial disruption, decreased vascular tone, and increased vascular permeability [28, 29].

For many years, it has been appreciated that critically ill patients, including those with sepsis, routinely have very low plasma vitamin C concentrations (<15 μmol/L) [30–34]. Even when circulating concentrations of vitamin C reflect adherence to recommended dietary intake (~50 μmol/L) [35], the activation of complement-mediated inflammation may lead to inadequate intracellular concentrations [36]. In animal models of sepsis, intravenous repletion of vitamin C improved arteriolar responsiveness to vasoconstrictors and capillary blood flow and decreased microvascular permeability and organ dysfunction [37–39]. In models of lung injury, vitamin C improved epithelial barrier function and alveolar fluid clearance and attenuated microvascular coagulation abnormalities and thrombosis in the lung [40, 41]. In a phase I study of patients with sepsis, pro-inflammatory markers were lower in patients who received intravenous vitamin C, as was thrombomodulin, a measure of endothelial injury [42]. In another phase I study of patients with vasopressor-dependent sepsis, norepinephrine dose and duration and patient mortality were all significantly lower among patients who received high-dose intravenous vitamin C [43].

A recent large observational study found neurological dysfunction to be the organ dysfunction most closely associated with early and late mortality among patients with sepsis [44]. Thus, interventions that are potentially neuroprotective should also be of interest. Vitamin C is one such therapy because of its antioxidant properties and its effects on the endothelium and blood–brain barrier. In an animal model of sepsis, treatment with antioxidants prevented the development of cognitive deficits at 30 days [45]. Furthermore, it has been observed that patients with sepsis and low concentrations of vitamin C in the plasma and cerebrospinal fluid more often exhibit encephalopathy, suggesting compromise of the blood–brain barrier [46, 47].

Like vitamin C, thiamine is an essential micronutrient that is frequently low in patients with sepsis [48]. Thiamine deficiency disrupts aerobic metabolism and, if severe, can lead to lactic acidosis and death. Furthermore, patients with septic shock and thiamine deficiency were recently demonstrated to clear lactate more quickly when given thiamine compared with similar patients who received placebo [49]. Moreover, they exhibited lower mortality. Lastly, thiamine modifies the metabolism of vitamin C so that less oxalate is generated, thereby decreasing the likelihood of oxalate nephropathy [50, 51].

Hydrocortisone for relative adrenal insufficiency among patients with sepsis has been studied in randomized controlled trials and in systematic reviews [52–57]. Although these studies provide inconsistent results with regard to the effect of hydrocortisone on mortality, corticosteroids are generally well tolerated and have been suggested to work synergistically with vitamin C to modify inflammatory mediators, increase catecholamine synthesis, improve endothelial function, and increase vasopressor sensitivity [17, 58–64]. The extent to which these interactions translate to a mortality benefit in the setting of sepsis is not known.

The purpose of this clinical trial is to test the efficacy of vitamin C, thiamine, and hydrocortisone as a combined therapy in patients with respiratory or circulatory dysfunction (or both) resulting from sepsis. These patients, at the critical end of the sepsis spectrum, have been chosen because they are easily identified, have high mortality, consume significant critical care resources, and endure significant reductions in physical, emotional, and cognitive quality of life if they survive. Any improvements in outcomes attributed to effective therapies would be of great value to patients as well as care providers and health-care systems. Furthermore, because this therapy is composed of three inexpensive and readily available drugs, its efficacy would have immediate and dramatic implications in the management of sepsis in both well and poorly resourced settings worldwide.

## Administrative responsibilities and relationships

**Fig. 1:**
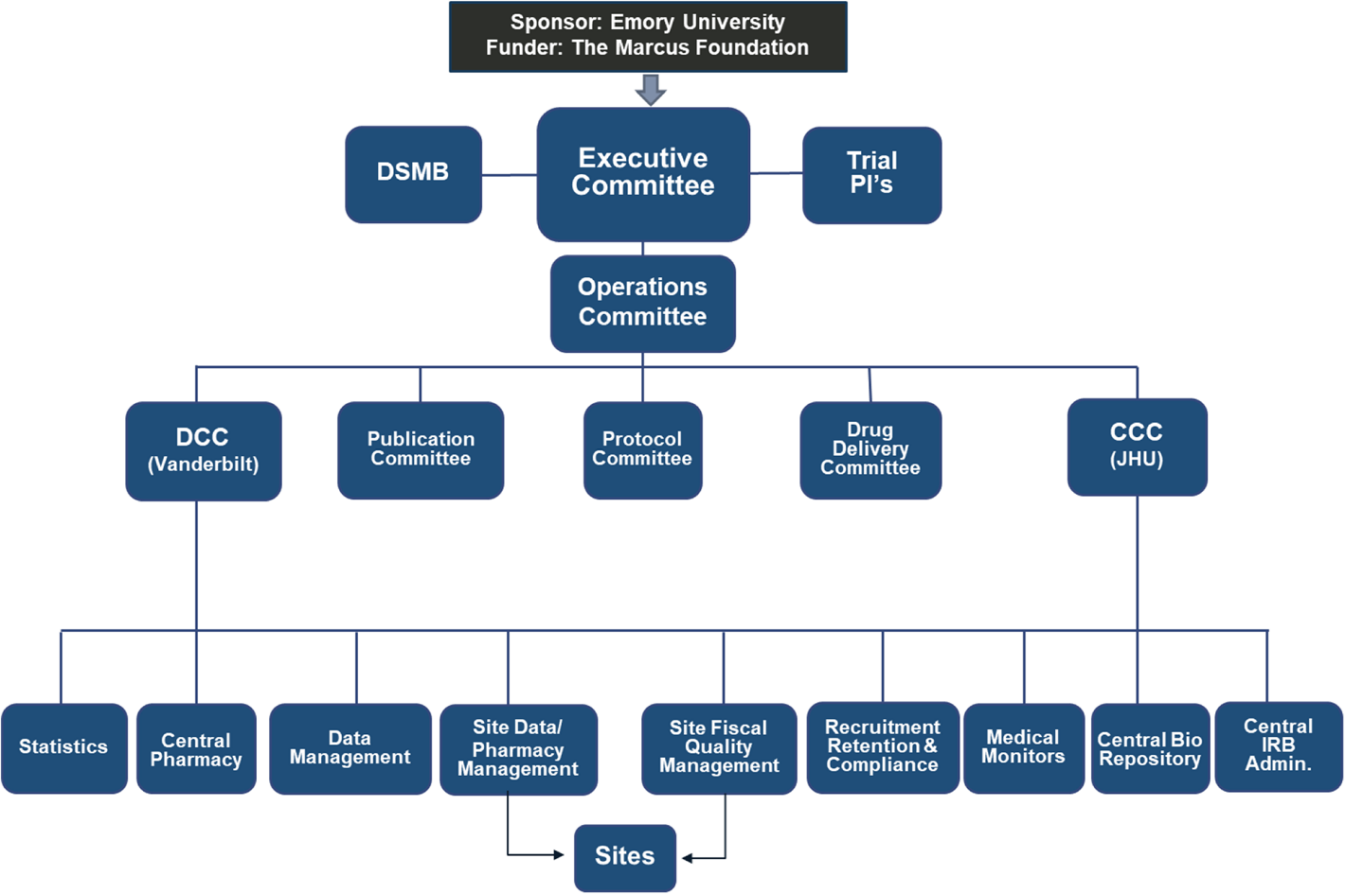
Administrative organization of study. Abbreviations: *CCC* Clinical Coordinating Center, *DCC* Data Coordinating Center, *DSMB* data safety monitoring board, *IRB* institutional review board, *JHU* Johns Hopkins University, *PI* principal investigator.

Funding for the Vitamin C, Thiamine and Steroids in Sepsis (VICTAS) study was extended by contract from a private foundation to Emory University, the sponsor of the study, with shared responsibility from principal investigators (PIs) at Emory University (JES, PI; DWW, co-PI) and Johns Hopkins University (RER, co-PI). The PIs subsequently recruited an executive committee and operations committee composed of experts in sepsis, clinical trial design, and trial execution. Subcommittees were created to develop the study protocol, plan for drug and placebo acquisition and delivery, construct a standard operating procedure for the use of data acquired during the study, and oversee the writing, authorship, and dissemination of study-related manuscripts (Additional file 1). Clinical Coordinating Center (CCC) and Data Coordinating Center (DCC) responsibilities were delegated to Johns Hopkins University and Vanderbilt University Medical Center, respectively (Fig. 1). Briefly, the CCC was assigned the responsibility of shepherding the trial protocol through the central institutional review board (cIRB) located at Johns Hopkins University, recruiting and onboarding about 40 participating sites, monitoring protocol adherence, and organizing and maintaining a biorepository. The DCC developed and will maintain the electronic system for data capture (Research Electronic Data Capture, or REDCap) and will conduct final statistical analyses. The central coordinating pharmacy will also be located at Vanderbilt University Medical Center.

## Methods

The VICTAS trial protocol was approved by the Johns Hopkins cIRB (IRB00164053). Any sites participating in the trial must formally agree to rely on this cIRB mechanism.

### Study design

The VICTAS trial is a prospective, multi-center, double-blind, adaptive sample size, randomized, placebo-controlled, clinical trial designed to investigate the efficacy of vitamin C, thiamine, and hydrocortisone (hereafter “treatment protocol” or “TP”) versus indistinguishable placebos (hereafter “control protocol” or “CP”) on the outcomes of patients with sepsis. Note that patients randomly assigned to the CP will be permitted to receive open-label steroids if prescribed by the treating clinical team. As such, patients randomly assigned to the CP compose a “comparator group” rather than a purely placebo group. Enrolled patients will be randomly assigned in a 1:1 ratio to the TP or CP.

### Population/Setting

Patients will be recruited from about 40 academic and non-academic medical centers in the United States. Any patient admitted to a study site and diagnosed with sepsis or septic shock with associated respiratory or cardiovascular dysfunction (or both) will be considered for enrollment. Subjects must meet all inclusion criteria and no exclusion criteria at the time of randomization, which must occur within 24 h of the recognized onset of sepsis-related respiratory or cardiovascular organ dysfunction. Specific inclusion and exclusion criteria are as follows:

### Inclusion criteria

Each of the following criteria must be met by a patient before enrollment can be considered:

- suspected or confirmed infection as evidenced by the ordering of blood cultures and the administration of at least one antimicrobial agent
- anticipated or confirmed ICU admission
- acute respiratory or cardiovascular organ dysfunction (or both) attributed to sepsis characterized by at least one of the following at the time of randomization:
  - ◦ respiratory support requirement: acute hypoxemic respiratory failure defined as persistent hypoxemia—partial pressure of oxygen/fraction of inspired oxygen (PaO_2_/FiO_2_) of not more than 300 or blood oxygen saturation/FiO_2_ (SpO_2_/FiO_2_) of not more than 315—requiring (1) intubation and mechanical ventilation, (2) non-invasive positive pressure ventilation (NIPPV) via a tight-fitting face mask, or (3) high-flow nasal cannula of at least 40 L per minute with an FiO_2_ of at least 0.40.
  - ◦ vasopressor requirement: a continuous infusion of norepinephrine, epinephrine, vasopressin, dopamine, phenylephrine, or other vasopressor agent at any dose for more than 1 h and required to maintain a mean arterial pressure of at least 65 mm Hg despite intravenous crystalloid resuscitation of at least 1000 mL.

### Exclusion criteria

A patient meeting any of the following criteria may not be enrolled:

- age of less than 18 years
- prior enrollment in VICTAS
- qualifying organ dysfunction no longer present at the time a subject would be randomly assigned (i.e., does not require either (1) respiratory support as defined above to maintain PaO_2_/FiO_2_ of more than 300 or SpO_2_/FiO_2_ of more than 315 or (2) vasopressor infusion to maintain a mean arterial pressure of at least 65 mm Hg)
- cardiovascular or respiratory organ failure caused by an illness other than sepsis
- first episode of qualifying organ dysfunction during a given emergency department (ED) or ICU admission occurred more than 24 h before subject could be randomly assigned
- limitations of care (defined as refusal of cardiovascular and respiratory support modes described in inclusion criteria), including “do not intubate” status
- current hospitalization of more than 30 days at the time the subject is considered for enrollment
- chronic hypoxemia requiring supplemental non-invasive oxygen via nasal cannula or NIPPV (i.e., continuous positive airway pressure and bi-level positive airway pressure) or home mechanical ventilation
- chronic cardiovascular failure requiring home mechanical hemodynamic support (e.g., ventricular assist device) or home chemical hemodynamic support (e.g., milrinone)
- known allergy or known contraindication to vitamin C, thiamine, or corticosteroids (including previous history or active diagnosis of primary hyperoxaluria or oxalate nephropathy or both, known/suspected ethylene glycol ingestion, or known glucose-6-phosphate dehydrogenase deficiency)
- use of vitamin C at a dose of greater than 1 g daily (oral of intravenous) within the 24 h preceding first episode of qualifying organ dysfunction
- chronic disease/illness that, in the opinion of the site investigator, has an expected life span of less than 30 days unrelated to current sepsis diagnosis (e.g., stage IV malignancy and neurodegenerative disease)
- pregnancy or known active breastfeeding
- prisoner or incarceration
- current participation in another interventional research study (Note: Co-enrollment in other interventional research studies may be considered but will require *a priori* written permission from the VICTAS executive committee in advance of subject identification.)
- inability or unwillingness of subject or legal surrogate/representative to give written informed consent.

### Screening and consent

Participating sites will be required to develop site-specific screening procedures to ensure the timely identification of potentially eligible patients. A brief summary of these procedures will be submitted to the CCC for review prior to site activation. All patients meeting study inclusion criteria will be recorded in the VICTAS screening log housed in REDCap at the DCC. Logs will be updated daily and “none” will be indicated if no patients meet inclusion criteria on a given calendar day. The CCC will review each site’s screening log at least once a month to identify the numbers of patients screened, those approached for consent, and the reason any patient may have been excluded.

Screen failures will include patients meeting all enrollment criteria who are not identified in a timely way, those who decline to participate, those who are not approached because either they or their legally authorized representative (LAR) cannot be reached, those who consent to enrollment but are found to violate eligibility criteria prior to randomization, and those who are not approached because of the presence of any other exclusion criteria.

Registered and credentialed study personnel will obtain consent directly from eligible patients with preserved capacity. When patients are not deemed capable of informed consent, the LAR will be approached as allowed by institutional standards and state requirements. When consent is obtained from an LAR, attempts to verify and obtain written consent from each patient for continued study participation is to be assessed regularly during his or her hospitalization. If consent is denied or withdrawn by either the enrolled participant or LAR, the participant will be withdrawn from the study.

In addition to consenting to be randomly assigned to the TP or CP, enrolled subjects will be asked to “opt in” and (1) contribute to the establishment of a biorepository (accrued from a subset of participating sites) to assess inflammatory markers of sepsis and to establish a bank of specimens for future studies and (2) partake in an assessment of neurocognitive long-term outcomes (LTOs) which focuses primarily on cognitive and psychological functioning. This battery, composed of tests that are appropriate to employ via telephone, is relatively brief (~35 min) but rigorous and samples the various domains of functioning known to be affected by critical illness and sepsis [65]. Participation in the biorepository and LTO assessments is not required for participation in the primary study.

### Randomization

Participants will be randomly assigned to the TP or CP in a 1:1 ratio. The randomization sequence will be generated in R (version 3.4.3, R Foundation, Vienna, Austria) using permuted small blocks of random size, stratified by site. Study arm allocation will be operationalized via the use of pre-sorted drug kits. Once consent is obtained and inclusion and exclusion criteria are verified through the online REDCap portal, participants will be assigned the next drug kit in the sequence. Participants will be considered enrolled in the trial when assigned to a drug kit.

### Investigational new drug exemption, study drugs, drug distribution, and storage

The VICTAS trial received an Investigational New Drug exemption from the US Food and Drug Administration (FDA) in January 2018 on the basis of the off-label use of FDA-approved medications that meet the regulatory criteria for exemption (21 CFR Sec. 312.2(b) (1)). All three products (vitamin C, thiamine, and hydrocortisone) are commercially available and were purchased from McGuff Pharmaceuticals, Inc. (Santa Ana, CA, USA). The central coordinating pharmacy at Vanderbilt University Medical Center will receive all study drugs and matching placebos in their commercial formulation and packaging. They will then be labeled as investigational product, coded with unique identification numbers, and packaged into study kits. Contents for each kit (TP or CP) will be determined by the randomization sequence. Study kits will be labeled using unique, ordered kit numbers. Numbered kits then will be shipped to investigational pharmacists at participating VICTAS sites. Each kit contains two boxes, both labeled with the kit number. Box “F” contains vitamin C or placebo and requires refrigeration at 2–8 °C. Box “RT” contains thiamine hydrochloride and hydrocortisone sodium succinate or matching placebos and will be stored at ambient temperature (20–25 °C). Upon unsealing boxes F and RT, site pharmacists will be unblinded to study arm allocation but are prohibited by protocol from informing anyone of study drug assignment.

### Intervention

Randomly assigned subjects will receive either study drugs or matching placebos. All study drugs and placebos will be administered intravenously every 6 h until a total of 16 administrations have occurred over 96 h or the patient is discharged from the ICU, whichever occurs first. Active agents include vitamin C (1.5 g), thiamine hydrochloride (100 mg), and hydrocortisone sodium succinate (50 mg). Patients will receive the first dose of study drugs, or placebos, within 4 h of randomization. All subsequent doses should be given every 6 h according to the standard every 6 h medication administrtion schedule used at each participating site. All drugs are to be administered separately and should not be infused simultaneously through the same line with any other medications. Thiamine and hydrocortisone will each be administered as an intravenous push, and vitamin C will be administered as a 30-min infusion. The same procedure will be followed for matching placebos. In patients who receive open-label corticosteroids by the clinical team at a total daily dose of at least 200 mg of hydrocortisone (or equivalent), hydrocortisone or matching placebo will be withheld by the investigational pharmacy. If the clinical team discontinues open-label steroids, hydrocortisone or placebo will resume until the patient completes the 96-h intervention period or the patient is discharged from the ICU.

Other than the administration of study drugs, all management of randomly assigned patients will be at the discretion of the clinical team and according to local protocols. This includes fluid resuscitation, antibiotics, vasopressor titration, mechanical ventilation and ventilator weaning strategies, blood transfusion, nutrition, renal replacement therapy, and delirium management.

Although glycemic control will also be managed by the clinical team and local protocols, it has been demonstrated that many point of care (POC) glucometers generate falsely elevated readings in the setting of high concentrations of intravenous vitamin C [66–69]. To avoid the possibility of missing clinically important hypoglycemia or inadvertently causing hypoglycemia (with inappropriate insulin), participating sites will be required to measure glucose using either central or critical care laboratory devices or a POC device that has been validated in the setting of high plasma concentrations of vitamin C [70].

### Data collection

**Fig. 2:**
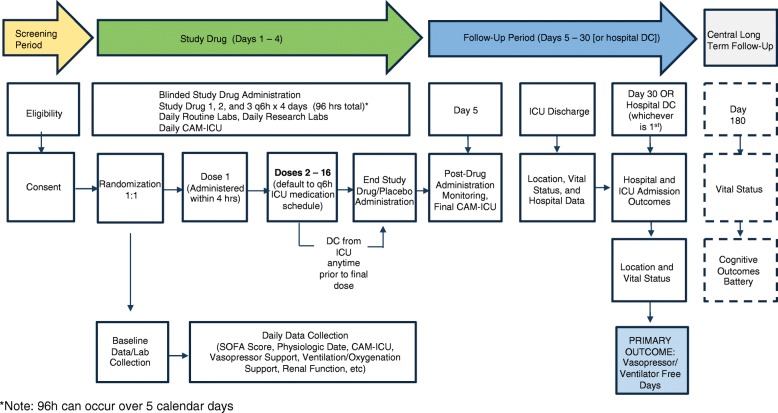
Overview of study progression. Abbreviations: *CAM-ICU* Confusion Assessment Method for the Intensive Care Unit, *DC* discharge, *ICU* intensive care unit, *SOFA* Sequential Organ Failure Assessment.

Enrolled patients will be evaluated clinically and by laboratory assessment at the time of randomization, on days of study drug or placebo infusion, at ICU discharge, and at hospital discharge or day 30, whichever occurs first (Table 1 and Fig. 2). Baseline data will be obtained as close as possible to the time of randomization and will include patient demographics, anthropometrics, source of hospital and ICU admission, health-care location (ED or ICU), comorbid conditions, presumed source of infection, antibiotic therapy, vital signs, level of respiratory support, vasopressor use, and clinical laboratory data when available and as needed to calculate the Acute Physiology and Chronic Health Evaluation II (APACHE II) and Sequential Organ Failure Assessment (SOFA) scores [71, 72]. Additionally, sedation level according to the Richmond Agitation and Sedation Scale (RASS) will be recorded, as will the presence or absence of delirium according to the Confusion Assessment Method for the Intensive Care Unit (CAM-ICU) [73–75]. On subsequent calendar days, clinical and laboratory data from 8 a.m. (or as close as possible to 8 a.m.) will be collected according to the schedule of events (Table 1).

**Table 1:** Schedule of events

Events	Screen enroll (time of randomization)	Day 1	Day 2	Day 3	Day 4	Day 5	ICU D/C	Hospital D/C	Day 30	Day 180
Procedure										
Eligibility Verification	X									
Informed consent	X									
Randomization	X									
Study drug admin^a^	X	X	X	X	X	X				
Demographics	X									
Anthropometrics	X									
Source of Admission	X									
History and physical^b^ (including comorbidity)	X									
Respiratory support^b^	X	X	X	X	X	X	X	X	O	
Vasopressor use (each agent and dose)^b,c^	X	X	X	X	X	X	X	X	O	
APACHE II^d^ [71]	X									
SOFA score^d^ [72]	X	X	X	X	X	X				
Vitals^e^	X	X	X	X	X	X				
GCS [90]	X	X	X	X	X	X				
RASS [73]	X	X	X	X	X	X				
CAM-ICU^f^ [74, 75]	X	X	X	X	X	X				
Hematology (platelets)	A	A	A	A	A	A				
Chemistry (T. bili, creatinine)	A	A	A	A	A	A				
Lactate	A	A	A							
Coagulation	A									
Pregnancy test^g^	X									
Central research labs^h^	X	X	X	X	X					
Antimicrobial therapy^b^	X						X	X	O	
Infection source data^b^	X						X	X		
Health-care location	X	X	X	X	X	X	X	X	O	C
Adverse event monitoring										
Potentially associated	X	X	X	X	X	X	X	X	O	
Serious	X	X	X	X	X	X	X	X	O	
Subject completion and follow-up										
Vital status							X	X	O	C
Renal Replacement free days								X	O	
VVFD								X	O	
Neuro-psychological Battery [65]										C

X = Performed by study site

A = Collect if available

O = Performed by study site only if patient remains hospitalized at 30 days

C = Performed by Central Long-Term Outcomes Team

^a^Note: patients who receive not more than three administrations of study drug/placebo on calendar day 1 will complete the last dose(s) on calendar day 5 (if they remain in the intensive care unit (ICU) that long)

^b^Will be abstracted from electronic medical record (EMR). Abstracted data will include baseline data and daily data. For baseline data, use data from as close to the time of randomization as is possible. For daily values, use data from as close to 8 a.m. as is possible up to day 5 or ICU discharge (whichever occurs first). Vasopresssor doses will be recorded only at time of randomization

^c^After day 5 or ICU discharge (whichever occurs first), report only the use of vasopressors or not (yes/no)

^d^Data elements collected via Research Electronic Data Capture (REDCap), score calculated centrally. For baseline Acute Physiology and Chronic Health Evaluation II (APACHE II), use most aberrant elements from the 24 h preceding the time of randomization. For baseline Sequential Organ Failure Assessment (SOFA) score, use data elements as close as possible to and preceding the time of randomization. For daily SOFA scores, use data elements from as close to 8 a.m. as is possible up to day 5 or ICU discharge (whichever occurs first)

^e^Mean arterial pressure, heart rate, respiratory rate, and temperature will be abstracted from EMR. Abstracted data will include baseline data and daily data. For baseline data, use data from as close to the time of randomization as is possible. For daily values, use data from as close to 8 a.m. as is possible up to day 5 of ICU discharge (whichever occurs first)

^f^Performed by research staff, at time of randomization and days 1–5 or ICU discharge (whichever occurs first)

^g^Pregnancy test (serum or urine), documentation of surgical sterilization, or menopausal required for eligibility. If not performed as standard of care, patient will not be eligible

^h^Central research labs will be collected at designated sites only.

Abbreviations: *CAM-ICU* Confusion Assessment Method for the Intensive Care Unit, *D/C* discharge, *GCS* Glasgow Coma Scale, *RASS* Richmond Agitation and Sedation Scale, *T. bili* total bilirubin, *VVFD* ventilator- and vasopressor-free day

For patients contributing to the biorepository, blood and urine samples will be obtained immediately prior to the first doses of study drugs or placebos on calendar day 1 of the study and 30 min after the first administrations are complete. Provided that the patient remains in the ICU, one additional collection of blood and urine per day will occur within 1 h prior to any study drug or placebo administration on calendar days 2 through 4. All specimens collected will be de-identified and associated with each patient by using a unique identifier.

Patients who agree to participate in the LTO sub-study will be contacted by phone at 180 days following randomization and undergo a validated and sensitive telephone-based neurocognitive assessment [65]. We will rely on well-established methods honed across other studies of cognitive outcomes in ICU survivors to ensure high follow-up rates and patient engagement [76]. All neurocognitive assessments will be carried out by neuropsychology coordinators at the Vanderbilt University LTO Coordinating Center to ensure consistency in approach and administration quality.

### Outcomes

The primary outcome for this trial is the number of consecutive days free of both ventilator and vasopressor support (VVFD) during the 30 days following randomization, recorded to the nearest calendar day. Patients who die at any time during the 30-day window score zero VVFDs. Patients who return to ventilator or vasopressor support will have the VVFD count reset to zero days so that ventilator- and vasopressor-free days accrue only from the last day the patient was free of both ventilator (including NIPPV and high-flow nasal cannula as per enrollment criteria) and vasopressor support. There are two mechanisms by which VVFDs may be increased among patients randomly assigned to the TP relative to the CP. First, the TP may reduce deaths. Second, it may reduce the number of days spent on respiratory or cardiovascular support among those subjects who do not die. Thus, there may be benefit in mortality, speed of recovery, or both, each translating to an increase in VVFDs.

The key secondary short-term outcome is 30-day mortality. Additional short-term outcomes to support efficacy include ICU mortality and ICU and hospital length of stay. Exploratory outcomes include ICU delirium and renal replacement–free days at day 30. Any changes in SOFA score during the 96-h intervention period will be characterized. Blood and urine specimens from the VICTAS biorepository will be used to characterize (1) baseline levels of vitamin C and the pharmacokinetics of vitamin C during sepsis, (2) biomarker signals associated with sepsis progression and response to therapy, including procalcitonin, C-reactive protein, and F2 isoprostane (a reliable and sensitive biomarker of oxidative stress), and (3) the performance of emerging molecular technologies for sepsis diagnosis and prognostication.

Important LTOs at 180 days following randomization include vital status and neurocognitive status among survivors. Neurocognitive status will be characterized with a wide array of standardized telephone-based cognitive, mental health, and functional assessments as described previously (Table 2) [77–87].

**Table 2:** Long-term outcomes assessments*

	Domain	Test name
Cognition	Attention	Attention (WAIS-IV Digit Span) [77] and delirium (Telephone Confusion Assessment Method) [78]
	Executive function	Executive functioning (Hayling Test) [79]
	Language	Language (Controlled Oral Word Association Test or COWA) [80]
	Memory	Memory (Paragraph Recall from the WMS-IV) [82]
	Orientation	Orientation (Telephone Interview for Cognitive Status) [81]
	Reasoning	Reasoning (WAIS-IV Similarities) [77]
Functioning	Basic and high-order functioning	Activities of daily living (Katz ADL) [83] Employment (Employment Questionnaire) and instrumental activities of daily living (Functional Activities Questionnaire) [84]
Mental health	Depression PTSD	Depression (Beck Depression Inventory-II) [85] and PTSD (Post-Traumatic Stress Disorder Checklist for the DSM-V)
Quality of life	General quality of life	EuroQol, 5 dimension (EQ5D) [87]

Abbreviations: *DSM-V* Diagnostic and Statistical Manual of Mental Disorders Version 5, *EQ5D* European Quality of Life Scale Five Dimensions, *EuroQol* European Quality of Life Scale, *WAIS-IV* Wechsler Adult Intelligence Scale Version 4, *WMS-IV* Wechsler Memory Scale Version 4

*All long-term outcomes will be conducted using valid and sensitive telephone-based neurocognitive assessments [65]

### Adverse event reporting

For the purposes of this study, adverse events (AEs) and serious AEs (SAEs) are defined in accordance with the guidelines of the US Office for Human Research Protections [88]. An AE is “any untoward medical occurrence in a human subject, including any abnormal sign (e.g., abnormal physical exam or laboratory finding), symptom, or disease” and occurs during a subject’s participation in research [88]. Expected AEs are those that are anticipated in the population under study, regardless of participation in research. Examples of expected AEs in this study include respiratory failure, thromboembolic disease, arrhythmias, delirium, anemia, coagulopathy, hypoglycemia, and death. Both expected and unexpected AEs will be considered study-related events, and thus reportable, if they are thought by study investigators to be related to study procedures or lead to discontinuation of study interventions.

Because it is not always clear that an AE is related to research, potentially associated AEs (PAAEs) are defined as those that could be related to research procedures. SAEs are those that occur following randomization up to the time of hospital discharge or day 30 (whichever occurs first) and fulfill any of the following criteria:

1. results in death
2. is life-threatening
3. results in prolongation of the existing hospitalization
4. results in a persistent or significant disability/incapacity
5. results in a congenital anomaly/birth defect or
6. is determined to be an important and significant medical event that could jeopardize the subject’s health and could require medical or surgical intervention to prevent one of the five outcomes listed above.

All unexpected or research-related AEs and SAEs as well as PAAEs will be reported to the DCC electronically through the REDCap system. AEs and PAAEs will be summarized at quarterly intervals for data safety monitoring board (DSMB) review and as needed for IRB continual renewals. Unexpected SAEs that are determined to be definitely or possibly related to the study will be reported by the site investigator to the DCC within 72 h. These events will be reviewed by the DCC and may be reclassified as PAAEs or—if confirmed as a true unexpected, related SAE—forwarded with supporting materials to an independent medical monitor at the CCC for further review. Events that are deemed to be unexpected SAEs and definitely or possibly related to the study will be reported to the DSMB chair as required by the DSMB charter. All unexpected related SAEs will also be reported to the cIRB at Johns Hopkins University in accordance with reporting requirements and may also be reported to participating sites if required.

In the event of a significant safety concern related to study drug administration, including any unanticipated drug interactions, the site PI should evaluate the situation and determine with the clinical team whether discontinuing the study drugs is warranted. Because there are no specific antidotes for vitamin C, thiamine, and hydrocortisone, simply discontinuing the study drugs is appropriate. However, the study medication blind will not be broken, as doing so will not provide increased safety.

### Data safety monitoring board

Data and safety monitoring will be conducted by an independent DSMB to ensure and maintain the scientific integrity and ethical balance of human subjects’ research and to protect subjects from avoidable harm. As detailed in its charter, the VICTAS trial DSMB will be composed of five individuals: two emergency medicine clinicians, two critical care clinicians, and one statistician. These individuals will be selected on the basis of their content expertise in sepsis, critical care, multi-center clinical trials, and adaptive trial design and implementation. The DSMB will meet at least twice a year until study completion and will report to the VICTAS executive committee. The DSMB will act independently of the funder and sponsor of the study and is charged with ensuring that the trial is implemented as designed and that the pre-specified design continues to be scientifically and ethically appropriate, and the DSMB will review ongoing safety data.

### Data management/monitoring

All data, unredacted source documents, and regulatory documents will be uploaded to REDCap and securely maintained by the DCC in electronic form. The REDCap application supports remote, centralized monitoring of participant data with an integrated query process. Specific data points that support the enrollment of participants or patient safety or affect the outcomes of interest (e.g., inclusion/exclusion criteria, informed consent, SAEs, and vasopressor/ventilator-free days) will be thoroughly reviewed by a study monitor. The first and tenth participant at each site will be 100% monitored for accuracy. About 10–20% of all additional data points will be randomly monitored for accuracy. For any omitted data or data found to be inaccurate or inconsistent with the provided source or not backed up by a source record, the DCC will issue a query to the participating site. All such queries will be tracked by using a standardized data resolution workflow module in REDCap. In the event that a given site has several instances of non-compliance, a VICTAS project manager will conduct an on-site visit to create a corrective action plan. In addition, at the time of reporting and analyses, further verification of data will occur through the application of range (to confirm entered values are clinically possible values) and consistency (to confirm internal consistency) checks to the dataset. Any new queries will be resolved as for monitor-identified queries.

All users of the REDCap system will receive required training applicable to each individual’s role in the conduct of the trial (e.g., data entry, uploading source, and regulatory documents). The completion of this training will be tracked and attested to by trainees, who at the same time will verify their commitment to the appropriate conduct of the trial and expectations for data accuracy and security.

### Interim analysis and sample size selection (stopping rules)

In the absence of phase II data to guide estimates, this trial uses an adaptive approach to determine sample size. To maximize the possibility of detecting a benefit if it exists, the study is powered to detect a moderate effect on VVFDs with a maximum enrollment of 2000 subjects while allowing the trial to stop early if a large VVFD or mortality benefit is observed. Interim analyses will be conducted by statisticians with expertise in adaptive design who will be provided the data needed by the DCC. Then the results of these analyses, along with the design-specified actions, will be provided to the DSMB. The DSMB will communicate these actions, along with any safety or study conduct recommendations, to the VICTAS executive committee.

Early interim analyses will occur when 200, 300, and 400 subjects have been enrolled. Later interim analyses will occur when 500, 1000, and 1500 subjects have been enrolled. At each interim analysis, all data from completed subjects will be used, and Bayesian predictive distributions will be used to impute the outcomes for those with incomplete data. At early (*N* <500) and late (*N* ≥500) interim analyses, the following predictive prabability (PP) will be computed:

- success on the mortality outcome if all currently enrolled subjects are followed to completion (*PP*_*mort, current N*_)

At later interim analyses (*N* ≥500), the following additional predictive probabilities will be computed:

- success on the primary VVFD outcome if all currently enrolled subjects are followed to completion (*PP*_*VVFD*, *Current N*_)
- success on the primary VVFD outcome if the trial enrolls the maximum number of subjects (*PP*_*VVFD*, *Max N*_)
- success on the mortality outcome if the trial enrolls the maximum number of subjects (*PP*_*mort*, *Max N*_).

At early interim analyses, when 200, 300, and 400 subjects have been enrolled, if the predictive probability of finding a significant difference (with one-sided alpha set at 0.001) between study groups on mortality with the current number of subjects exceeds 90%, study accrual will be stopped for success, data collection on enrolled patients will be completed, and formal outcomes assessments will be conducted. There is no stopping rule for futility if fewer than 500 patients are enrolled.

At later interim analyses, when 500, 1000, and 1500 patients have been enrolled, if the predictive probability of success on VVFDs if the trial were to enroll all 2000 subjects is less than 10%, the trial will be stopped for futility. If the predictive probability of success on both the VVFDs and mortality endpoints exceeds 95% on currently enrolled subjects, accrual will be stopped for expected success. If the predictive probability of success on VVFDs for currently enrolled subjects exceeds 95% but the predictive probability of success on mortality is less than 10% should the trial continue to enroll 2000 subjects, the trial will stop for expected success on VVFDs alone since detecting any mortality benefit is likely out of reach. If accrual is stopped for expected success due to either condition, the study will continue until all enrolled patients reach their VVFD and mortality endpoints, at which time the final analysis will be conducted. Thus, predictive probabilities are not used in the final analysis. We note that it is highly unlikely that we will stop the trial for success on mortality alone without also concluding success on VVFDs since VVFDs represent a combination of treatment effect on mortality (all deaths are recorded as zero VVFDs) as well as treatment effect on vasopressor and ventilator support dependence in survivors. These stopping rules are summarized in Table 3.

**Table 3:** Interim decision rules

Sample size	Interim decision	Condition for decision
N < 500	Futility	*May be recommended by DSMB*
	Expected success (mortality)	*PP*_mort_ (current N) > 0.90
	Continue	*PP*_mort_ (current N) < 0.90
N ≥ 500	Futility	*PP*_*VVFD*_ *(max N) < 0.10*
	Expected success (both endpoints)	*PP*_*VVFD*_ *(current N) > 0.95 AND PP*_*mort*_ *(current N) > 0.95*
	Expected success (VVFD only)	*PP*_*VVFD*_ *(current N) > 0.95 AND PP*_*mort*_ *(max N) < 0.10*
	Continue	*otherwise*

The overall type I error rate for the trial is controlled at 2.5% (one-sided), and the early interims, when 200, 300, and 400 patients have been enrolled, are designed to conservatively spend alpha so that 2.4% remains for the analysis of 500 or more enrolled patients (Table 4). With these parameters and a 20% estimated mortality benefit, which is conservative compared with the 32% observed by Marik et al. [17], the study is very likely (approximately 97% chance) to stop at or before 400 patients if this large mortality benefit is real. The power estimates used for sample size selection if the trial progresses to 500 subjects and beyond were determined through clinical trial simulation and take into account both the primary outcome of VVFDs and the secondary mortality outcome (Additional file 2).

**Table 4:** Alpha spend for interim analyses

Interim analysis	Alpha spend
*N* = 200	0.0002
*N* = 300	< 0.0001
*N* = 400	0.0003
N = 500	0.010
*N* = 1000	0.0026
*N* = 1500	0.0033

### Data analysis

A detailed statistical analysis plan will be submitted as an update. In brief, continuous variables characterizing each study group will be reported as means with standard deviations or medians with interquartile ranges. Categorical variables will be represented as frequencies and proportions. The primary outcome is VVFDs at 30 days, and 30-day mortality is the key secondary outcome. VVFDs and mortality will be tabulated by study group and presented graphically. The primary analysis will be performed after all enrolled subjects have completed follow-up. For subjects with missing data on the primary endpoint, a “last status carried forward” approach will be used. If a subject was last seen on ventilatory support (as detailed in inclusion criteria) or vasopressors or both, it will be assumed that the subject remained so at 30 days, and a value of zero VVFDs will be imputed. If the subject was last seen off respiratory support or vasopressors or both and is not known to be dead, it will be assumed that the subject remained so for the remainder of the 30-day period. A gatekeeping strategy is used to control type I error rate. If the trial stops for expected success on mortality after enrolling 200, 300, or 400 patients, the mortality outcome will be tested first using a chi-squared test with one-sided alpha of 0.001. If the mortality outcome is successful, VVFDs will be compared using a Wilcoxon rank-sum test with a one-sided alpha of 0.022. If the trial reaches *N* = 500 or more, indicating more moderate effects, the more sensitive VVFD outcome will be tested first using a Wilcoxon rank-sum test with a one-sided alpha of 0.022. The mortality outcome will be tested only if a significant difference is detected on VVFDs using a chi-squared test with a one-sided alpha of 0.024.

Additional outcomes are ICU mortality, mortality at 180 days, ICU delirium, renal replacement–free days at day 30, ICU and hospital length of stay, and physical, emotional, and cognitive outcomes at 180 days. Comparisons between study groups will be made using chi-squared tests or the Wilcoxon rank-sum test, as appropriate. These tests will all be two-sided with no adjustment for multiple comparisons, although we will report the chance of a type I error. In further exploratory analyses, comparisons of primary, secondary, and exploratory outcomes between groups will be modeled using, for example, logistic regression for binary outcomes and proportional odds models for ordinal outcomes. All primary, secondary, and other efficacy analyses will be based on the intent-to-treat dataset with no modifications to the intention-to-treat principle; subjects will be classified as randomly assigned. A per-protocol analysis set will be generated for exploratory analyses. Subjects in the per-protocol analysis set must meet all inclusion and no exclusion criteria, receive at least four doses of study drugs or placebos, and have no major protocol deviations. Protocol deviations will be ascertained prior to unblinding.

Our approach to using last observed status carried forward for estimating VVFD and mortality when they are not observed at 30 days ensures that we will not have missing data on the primary outcome and the key secondary outcome. For analyses involving modeling of these endpoints, multiple imputation techniques based on predictive mean matching will be used to overcome missingness in covariates. Exploratory and secondary outcomes may be missing. The set of patients with complete outcomes data will be included when comparing these between the TP and CP groups. We may also conduct sensitivity analyses by conservatively imputing missing outcomes.

## Protocol amendments

Protocol amendments, once approved by the cIRB, will be disseminated to participating sites and investigator teams by site managers at the CCC using direct communications as well as monthly webinars. In addition, updates will be posted to VictasTrialSites.org and ClinicalTrials.gov.

## Discussion

The VICTAS trial has been organized to assess the efficacy of a combined regimen of intravenous vitamin C, thiamine, and hydrocortisone in patients with respiratory and cardiovascular compromise that is attributed to sepsis. Efficacy will be defined by either an early mortality benefit or a significant increase in the primary endpoint of VVFDs among patients randomly assigned to the TP. Importantly, VVFDs may be increased by a reduction in deaths or a reduction in days dependent on respiratory and/or cardiovascular support or a combination of the two.

Other important outcomes of interest include ICU mortality, renal replacement–free days, ICU and hospital length of stay, ICU delirium, and changes in SOFA score over time. The VICTAS trial will also generate data to assess the relationship between sepsis and neurocognitive functioning and any potential mitigating effect the three-drug regimen may have on short- and long-term neurocognitive outcomes. Lastly, the trial will create a biorepository which will be used to characterize vitamin C pharmacokinetics and to measure standard and emerging markers of sepsis severity in general and in the setting of high concentrations of vitamin C.

The prevalence of sepsis worldwide and the high morbidity and mortality of this common syndrome speak to the importance of finding effective therapies. For this reason, the large mortality benefit reported by Marik et al.; the benign nature of vitamin C, thiamine, and hydrocortisone; and the fact that all three drugs are readily available and inexpensive have fueled an intense enthusiasm for this therapy. This has resulted in the initiation of several phase I and II trials and the VICTAS trial, which arguably fits the definition of a phase III trial. Although the initiation of a phase III trial without phase II data is atypical and considered financially risky, it is not without precedent, especially in the pharmaceutical industry where it may be stimulated by competition [89]. The VICTAS investigators are in the unique position of having been approached and supported by a funder who is determined to quickly assess the efficacy of the three-drug regimen and therefore has provided the resources necessary to organize and start the trial.

At the time of this writing, there are 17 randomized controlled clinical trials of the use of vitamin C in sepsis, in addition to the VICTAS trial, registered at ClinicalTrials.gov (accessed 10/4/2018). Of the four studies of vitamin C alone versus placebo that have been completed (NCT01590303, NCT01434121, NCT02734147, and NCT02106975), only one (NCT01434121), a study of pharmacokinetics and markers of inflammation, has been published [42]. Of the studies that are ongoing or planned, two compare vitamin C with placebo (NC03338569 and NCT03680274), one compares vitamin C and thiamine with placebos (NCT03592277), two compare vitamin C and hydrocortisone with placebos (NCT03592693 and NCT03649633), two compare the three-drug regimen with hydrocortisone alone (NCT03333278 and NCT03540628), four compare the three-drug regimen with matching placebos (NCT03258684, NCT03335124, NCT03422159, and NCT03389555), and one compares the three-drug regimen with usual care (NCT03380507). The largest of these studies plans to enroll 800 subjects (NCT03680274). VICTAS has an initial planned enrollment of up to 500 patients and will possibly enroll as many as 2000 (NCT03509350). In addition, to our knowledge, VICTAS is unique in that the three-drug regimen is compared with a group in which open-label steroids are permitted. As a result, patients will not be prevented by protocol from receiving stress-dose steroids if their clinical team feels this treatment is appropriate. It is recognized that this may bias the results toward the null given our primary endpoint of VVFDs. However, in studies that do not allow the use of steroids in the control population, it may be difficult to attribute improvements in outcomes to vitamin C as opposed to steroids.

An additional unique aspect of the VICTAS trial is the attention paid to neurocognitive outcomes. As has recently been reported, neurological dysfunction is the organ dysfunction most closely associated with early and late mortality among patients with sepsis and an extremely common and disruptive phenomenon among survivors of sepsis, even at extremely distal time points [44]. The assessments of delirium while patients are receiving study drugs or placebos in the ICU and the neurocognitive assessments at 180 days will give insight into the hypothesis that individuals experiencing “less” sepsis have fewer short- and long-term cognitive deficits. In addition, the value of the three-drug regimen as a preventative therapy for these outcomes will be assessed.

There are several limitations to this study, many of which are the result of limited phase II data. First, there are few previous data to guide sample size calculations. However, the use of interim analyses with pre-stated stopping rules allows the early recognition of a large mortality benefit, consistent with the recent observational pre-post data. In the absence of an early mortality benefit, the analysis refocuses on the more sensitive outcome of VVFDs to maximize the chances of observing an efficacy signal if one is present. Second, without phase II data, there is limited experience to guide the optimal dosing regimen for vitamin C. Therefore, a negative study could be consistent with an ineffective therapy or an ineffective dosing regimen. Although using the same total dose in all patients randomly assigned to the TP (as opposed to a weight-based dose) decreases pharmacy costs and simplifies the conduct of the trial, it will also result in some patients being exposed to substantially higher blood concentrations of vitamin C than others. Although the known adverse effects of vitamin C are rare and usually mild, the VICTAS dosing regimen may increase the likelihood of these events, especially in patients with lower body weights or impaired renal function. Third, owing to the inclusion of steroids in the TP, there is the potential for investigators and clinical providers to become unblinded because of otherwise unanticipated aberrations in serum glucose levels. Fourth, because the intervention group will receive the three-drug regimen, we will not be able to characterize which drug in the regimen is paramount. However, given the potential synergy between drugs, establishing the extent to which the combination affects outcomes has been prioritized. If a beneficial effect is observed, subsequent studies will be needed to determine the optimal dosing regimen and disentangle the effects of each component. Fifth, although the inclusion criteria for VICTAS are consistent with sepsis as defined by “The Third International Consensus Definitions for Sepsis and Septic Shock” (Sepsis-3) [1], they are also focused on respiratory and cardiovascular failure. Patients with sepsis based on abnormalities in other organ systems and without respiratory or cardiovascular organ dysfunction will not be enrolled. As such, the extent to which a beneficial finding can be applied to these subgroups of sepsis will not be known. However, regardless of this event, an individual patient meta-analysis of this and other contemporaneous trials (NCT03389555 and NCT03680274) comparing the use of intravenous vitamin C in patients with sepsis is planned (personal communication), increasing the likelihood that data obtained from patients enrolled in VICTAS will be informative. This protocol was developed in accordance with the SPIRIT 2013 guideline (Additional files 3 and 4) [91].

## Trial update

As of January 9, 2019, the VICTAS protocol version is 1.4. VICTAS began enrollment August 22, 2018. It is estimated that up to 500 subjects will be enrolled by September 2019. If enrollment continues to the maximum enrollment of 2000 subjects, the estimated completion date is December 2021.

## Additional files

Additional file 1: Trial publication and data management protocol. (PDF 293 kb)

Additional file 2: Adaptive design report. (PDF 3433 kb)

Additional file 3: World Health Organization Trial Registration Data. (XLSX 10 kb)

Additional file 4: SPIRIT 2013 Checklist: Recommended items to address in a clinical trial protocol and related documents. (DOC 122 kb)

## Abbreviations

AE: Adverse event
CCC: Clinical Coordinating Center
cIRB: Central institutional review board
CP: Control protocol
DCC: Data Coordinating Center
DSMB: Data safety monitoring board
ED: Emergency department
FDA: US Food and Drug Administration
FiO_2_: Fraction of inspired oxygen
ICU: Intensive care unit
IRB: Institutional review board
LAR: Legally authorized representative
LTO: Long-term outcome
NIPPV: Nasal intermittent positive pressure ventilation
PAAE: Potentially associated adverse event
PaO_2_: Partial pressure of oxygen
PI: Principal investigator
POC: Point of care
PP: Predictive probability
REDCap: Research Electronic Data Capture
SAE: Severe adverse event
SOFA: Sequential Organ Failure Assessment
SpO_2_: Blood oxygen saturation
TP: Treatment protocol
VICTAS: Vitamin C, Thiamine and Steroids in Sepsis
VVFD: Ventilator- and vasopressor-free day

## References

[1] Singer M, Deutschman CS, Seymour CW, Shankar-Hari M, Annane D, Bauer M (2016). The Third International Consensus Definitions for Sepsis and Septic Shock (Sepsis-3). JAMA.

[2] Martin GS, Mannino DM, Eaton S, Moss M (2003). The epidemiology of sepsis in the United States from 1979 through 2000. N Engl J Med.

[3] Angus DC, Linde-Zwirble WT, Lidicker J, Clermont G, Carcillo J, Pinsky MR (2001). Epidemiology of severe sepsis in the United States: analysis of incidence, outcome, and associated costs of care. Crit Care Med.

[4] Rhee C, Dantes R, Epstein L, Murphy DJ, Seymour CW, Iwashyna TJ (2017). Incidence and trends of sepsis in US hospitals using clinical vs claims data, 2009-2014. JAMA.

[5] Kempker JA, Martin GS (2016). The changing epidemiology and definitions of sepsis. Clin Chest Med.

[6] Liu V, Escobar GJ, Whippy A, Angus DC, Iwashyna TJ. Sepsis contributes to nearly half of all hospital deaths in the US. Am J Respir Crit Care Med. 2014;189:A2188.

[7] Torio CM, Moore BJ (2006). National inpatient hospital costs: the most expensive conditions by payer, 2013: statistical brief #204.

[8] Karlsson S, Ruokonen E, Varpula T, Ala-Kokko TI, Pettila V, Finnsepsis Study G (2009). Long-term outcome and quality-adjusted life years after severe sepsis. Crit Care Med.

[9] Yende S, Austin S, Rhodes A, Finfer S, Opal S, Thompson T (2016). Long-term quality of life among survivors of severe sepsis: analyses of two international trials. Crit Care Med.

[10] Dellinger RP, Levy MM, Rhodes A, Annane D, Gerlach H, Opal SM (2013). Surviving sepsis campaign: international guidelines for management of severe sepsis and septic shock, 2012. Intensive Care Med.

[11] Rhodes A, Evans LE, Alhazzani W, Levy MM, Antonelli M, Ferrer R (2017). Surviving sepsis campaign: international guidelines for management of sepsis and septic shock: 2016. Intensive Care Med.

[12] Sebat F, Musthafa AA, Johnson D, Kramer AA, Shoffner D, Eliason M (2007). Effect of a rapid response system for patients in shock on time to treatment and mortality during 5 years. Crit Care Med.

[13] Castellanos-Ortega A, Suberviola B, Garcia-Astudillo LA, Holanda MS, Ortiz F, Llorca J (2010). Impact of the surviving sepsis campaign protocols on hospital length of stay and mortality in septic shock patients: results of a three-year follow-up quasi-experimental study. Crit Care Med.

[14] Miller RR, Dong L, Nelson NC, Brown SM, Kuttler KG, Probst DR (2013). Multicenter implementation of a severe sepsis and septic shock treatment bundle. Am J Respir Crit Care Med.

[15] Liu VX, Morehouse JW, Marelich GP, Soule J, Russell T, Skeath M (2016). Multicenter Implementation of a Treatment Bundle for Patients with Sepsis and Intermediate Lactate Values. Am J Respir Crit Care Med.

[16] Kumar G, Kumar N, Taneja A, Kaleekal T, Tarima S, McGinley E (2011). Nationwide trends of severe sepsis in the 21st century (2000-2007). Chest.

[17] Marik PE, Khangoora V, Rivera R, Hooper MH, Catravas J (2017). Hydrocortisone, vitamin C, and thiamine for the treatment of severe sepsis and septic shock: a retrospective before-after study. Chest.

[18] Harris R (2017). Doctor turns up possible treatment for deadly sepsis. NPR.

[19] Harris R (2018). Can a cocktail of vitamins and steroids cure a major killer in hospitals? NPR.

[20] Exploring vitamin C therapy in sepsis.: Jesse & Julie Rasch Foundation; [Available from: http://raschfoundation.org/projects/could-an-apple-a-day-keep-sepsis-away/. Accessed 1 Dec 2018.

[21] Artenstein AW, Higgins TL, Opal SM (2013). Sepsis and scientific revolutions. Crit Care Med.

[22] Marshall JC (2014). Why have clinical trials in sepsis failed?. Trends Mol Med.

[23] Harris R (2018). Why the newly proposed sepsis treatment needs more study? NPR.

[24] Walter JM, Singer BD (2017). Vitamin C and sepsis: framing the postpublication discussion. Chest.

[25] Michelow IC, Alhinai Z, Dennehy PH (2017). Hydrocortisone, vitamin C and thiamine for sepsis: whither the ethics in research?. Chest.

[26] Kalil AC, Johnson DW, Cawcutt KA (2017). Vitamin C is not ready for prime time in sepsis but a solution is close. Chest.

[27] Levine M, Rumsey SC, Daruwala R, Park JB, Wang Y (1999). Criteria and recommendations for vitamin C intake. JAMA.

[28] Parihar A, Parihar MS, Milner S, Bhat S (2008). Oxidative stress and anti-oxidative mobilization in burn injury. Burns.

[29] Rudyk O, Phinikaridou A, Prysyazhna O, Burgoyne JR, Botnar RM, Eaton P (2013). Protein kinase G oxidation is a major cause of injury during sepsis. Proc Natl Acad Sci U S A.

[30] Prauchner CA (2017). Oxidative stress in sepsis: pathophysiological implications justifying antioxidant co-therapy. Burns.

[31] Borrelli E, Roux-Lombard P, Grau GE, Girardin E, Ricou B, Dayer J (1996). Plasma concentrations of cytokines, their soluble receptors, and antioxidant vitamins can predict the development of multiple organ failure in patients at risk. Crit Care Med.

[32] Schorah CJ, Downing C, Piripitsi A, Gallivan L, Al-Hazaa AH, Sanderson MJ (1996). Total vitamin C, ascorbic acid, and dehydroascorbic acid concentrations in plasma of critically ill patients. Am J Clin Nutr.

[33] Nathens AB, Neff MJ, Jurkovich GJ, Klotz P, Farver K, Ruzinski JT (2002). Randomized, prospective trial of antioxidant supplementation in critically ill surgical patients. Ann Surg.

[34] Marcus SL, Dutcher JP, Paietta E, Ciobanu N, Strauman J, Wiernik PH (1987). Severe hypovitaminosis C occurring as the result of adoptive immunotherapy with high-dose interleukin 2 and lymphokine-activated killer cells. Cancer Res.

[35] Dietary Reference Intakes for Vitamin C, Vitamin E, Selenium, and Carotenoids. Washington (DC) 2000. 25077263

[36] Padh H, Aleo JJ (1987). Activation of serum complement leads to inhibition of ascorbic acid transport. Proc Soc Exp Biol Med.

[37] Armour J, Tyml K, Lidington D, Wilson JX (2001). Ascorbate prevents microvascular dysfunction in the skeletal muscle of the septic rat. J Appl Physiol (1985)..

[38] Wu F, Wilson JX, Tyml K (2004). Ascorbate protects against impaired arteriolar constriction in sepsis by inhibiting inducible nitric oxide synthase expression. Free Radic Biol Med.

[39] Fisher BJ, Kraskauskas D, Martin EJ, Farkas D, Puri P, Massey HD (2014). Attenuation of sepsis-induced organ injury in mice by vitamin C. JPEN J Parenter Enteral Nutr.

[40] Fisher BJ, Seropian IM, Kraskauskas D, Thakkar JN, Voelkel NF, Fowler AA (2011). Ascorbic acid attenuates lipopolysaccharide-induced acute lung injury. Crit Care Med.

[41] Fisher BJ, Kraskauskas D, Martin EJ, Farkas D, Wegelin JA, Brophy D (2012). Mechanisms of attenuation of abdominal sepsis induced acute lung injury by ascorbic acid. Am J Physiol Lung Cell Mol Physiol.

[42] Fowler AA, Syed AA, Knowlson S, Sculthorpe R, Farthing D, DeWilde C (2014). Phase I safety trial of intravenous ascorbic acid in patients with severe sepsis. J Transl Med.

[43] Zabet MH, Mohammadi M, Ramezani M, Khalili H (2016). Effect of high-dose Ascorbic acid on vasopressor's requirement in septic shock. J Res Pharm Pract.

[44] Schuler A, Wulf DA, Lu Y, Iwashyna TJ, Escobar GJ, Shah NH (2018). The impact of acute organ dysfunction on long-term survival in sepsis. Crit Care Med.

[45] Barichello T, Machado RA, Constantino L, Valvassori SS, Reus GZ, Martins MR (2007). Antioxidant treatment prevented late memory impairment in an animal model of sepsis. Crit Care Med.

[46] Voigt K, Kontush A, Stuerenburg HJ, Muench-Harrach D, Hansen HC, Kunze K (2002). Decreased plasma and cerebrospinal fluid ascorbate levels in patients with septic encephalopathy. Free Radic Res.

[47] Barichello T, Fortunato JJ, Vitali AM, Feier G, Reinke A, Moreira JC (2006). Oxidative variables in the rat brain after sepsis induced by cecal ligation and perforation. Crit Care Med.

[48] Donnino MW, Carney E, Cocchi MN, Barbash I, Chase M, Joyce N (2010). Thiamine deficiency in critically ill patients with sepsis. J Crit Care.

[49] Donnino MW, Andersen LW, Chase M, Berg KM, Tidswell M, Giberson T (2016). Randomized, double-blind, placebo-controlled trial of thiamine as a metabolic resuscitator in septic shock: a pilot study. Crit Care Med.

[50] Hoppe B, Beck BB, Milliner DS (2009). The primary hyperoxalurias. Kidney Int.

[51] Sidhu H, Gupta R, Thind SK, Nath R (1987). Oxalate metabolism in thiamine-deficient rats. Ann Nutr Metab.

[52] Annane D, Sebille V, Charpentier C, Bollaert PE, Francois B, Korach JM (2002). Effect of treatment with low doses of hydrocortisone and fludrocortisone on mortality in patients with septic shock. JAMA.

[53] Sprung CL, Annane D, Keh D, Moreno R, Singer M, Freivogel K (2008). Hydrocortisone therapy for patients with septic shock. N Engl J Med.

[54] Venkatesh B, Finfer S, Cohen J, Rajbhandari D, Arabi Y, Bellomo R (2018). Adjunctive glucocorticoid therapy in patients with septic shock. N Engl J Med.

[55] Annane D, Renault A, Brun-Buisson C, Megarbane B, Quenot JP, Siami S (2018). Hydrocortisone plus fludrocortisone for adults with septic shock. N Engl J Med.

[56] Gibbison B, Lopez-Lopez JA, Higgins JP, Miller T, Angelini GD, Lightman SL (2017). Corticosteroids in septic shock: a systematic review and network meta-analysis. Crit Care.

[57] Rochwerg B, Oczkowski SJ, Siemieniuk RAC, Agoritsas T, Belley-Cote E, D’Aragon F, et al. Corticosteroids in sepsis: an updated systematic review and meta-analysis. Crit Care Med. 2018;46:1411–1420. 10.1097/CCM.0000000000003262 29979221

[58] Annane D, Pastores SM, Rochwerg B, Arlt W, Balk RA, Beishuizen A (2017). Guidelines for the diagnosis and management of critical illness-related corticosteroid insufficiency (CIRCI) in critically ill patients (Part I): society of critical care medicine (SCCM) and European society of intensive care medicine (ESICM) 2017. Crit Care Med..

[59] Marik PE, Pastores SM, Annane D, Meduri GU, Sprung CL, Arlt W (2008). Recommendations for the diagnosis and management of corticosteroid insufficiency in critically ill adult patients: consensus statements from an international task force by the American College of Critical Care Medicine. Crit Care Med..

[60] Dillon PF, Root-Bernstein RS, Lieder CM (2004). Antioxidant-independent ascorbate enhancement of catecholamine-induced contractions of vascular smooth muscle. Am J Physiol Heart Circ Physiol..

[61] Wilson JX (2009). Mechanism of action of vitamin C in sepsis: ascorbate modulates redox signaling in endothelium. Biofactors.

[62] Wilson JX (2013). Evaluation of vitamin C for adjuvant sepsis therapy. Antioxid Redox Signal..

[63] Han M, Pendem S, Teh SL, Sukumaran DK, Wu F, Wilson JX (2010). Ascorbate protects endothelial barrier function during septic insult: Role of protein phosphatase type 2A. Free Radic Biol Med.

[64] May JM, Harrison FE (2013). Role of vitamin C in the function of the vascular endothelium. Antioxid Redox Signal..

[65] Taichman DB, Christie J, Biester R, Mortensen J, White J, Kaplan S (2005). Validation of a brief telephone battery for neurocognitive assessment of patients with pulmonary arterial hypertension. Respir Res.

[66] Vasudevan S, Hirsch IB (2014). Interference of intravenous vitamin C with blood glucose testing. Diabetes Care.

[67] Cho J, Ahn S, Yim J, Cheon Y, Jeong SH, Lee SG (2016). Influence of vitamin C and maltose on the accuracy of three models of glucose meters. Ann Lab Med.

[68] Ceriotti F, Kaczmarek E, Guerra E, Mastrantonio F, Lucarelli F, Valgimigli F (2015). Comparative performance assessment of point-of-care testing devices for measuring glucose and ketones at the patient bedside. J Diabetes Sci Technol.

[69] Hager DN, Martin GS, Sevransky JE, Hooper MH (2018). Glucometry when using vitamin C in sepsis: a note of caution. Chest.

[70] DuBois JA, Slingerland RJ, Fokkert M, Roman A, Tran NK, Clarke W (2017). Bedside Glucose Monitoring-Is it Safe? A New, Regulatory-Compliant Risk Assessment Evaluation Protocol in Critically Ill Patient Care Settings. Crit Care Med.

[71] Knaus WA, Draper EA, Wagner DP, Zimmerman JE (1985). APACHE II: a severity of disease classification system. Crit Care Med.

[72] Vincent JL, Moreno R, Takala J, Willatts S, De Mendonca A, Bruining H (1996). The SOFA (Sepsis-related Organ Failure Assessment) score to describe organ dysfunction/failure. On behalf of the Working Group on Sepsis-Related Problems of the European Society of Intensive Care Medicine. Intensive Care Med.

[73] Sessler CN, Gosnell MS, Grap MJ, Brophy GM, O’Neal PV, Keane KA (2002). The Richmond Agitation-Sedation Scale: validity and reliability in adult intensive care unit patients. AmJ RespirCrit Care Med.

[74] Ely EW, Margolin R, Francis J, May L, Truman B, Dittus R (2001). Evaluation of delirium in critically ill patients: validation of the Confusion Assessment Method for the Intensive Care Unit (CAM-ICU). Crit Care Med.

[75] Ely EW, Inouye SK, Bernard GR, Gordon S, Francis J, May L (2001). Delirium in mechanically ventilated patients: validity and reliability of the confusion assessment method for the intensive care unit (CAM-ICU). JAMA.

[76] Pandharipande PP, Girard TD, Jackson JC, Morandi A, Thompson JL, Pun BT (2013). Long-term cognitive impairment after critical illness. N Engl J Med.

[77] Hartman DE (2009). Wechsler Adult Intelligence Scale IV (WAIS IV): return of the gold standard. Appl Neuropsychol.

[78] Marcantonio ER, Michaels M, Resnick NM (1998). Diagnosing delirium by telephone. J Gen Intern Med.

[79] Burgess PW, Shallice T (2007). The Hayling and Brixton Tests.

[80] Lezak MD (1995). Neuropsychological Assessment.

[81] Brandt JSM, Folstein MF. The Telephone Interview for Cognitive Status. Neuropsychiatry Neuropsychol Behav Neurol. 1988:111–7.

[82] Wechsler D (2009). WMS-IV Technical and Interpretive Manual.

[83] Katz S, Ford AB, Moskowitz RW, Jackson BA, Jaffe MW (1963). Studies of illness in the aged. The index of ADL: a standardized measure of biological and psychosocial function. JAMA.

[84] Pfeffer RI, Kurosaki TT, Harrah CH, Chance JM, Filos S (1982). Measurement of functional activities in older adults in the community. J Gerontol.

[85] Beck ATS, Steer RA, Brown GK (1996). Beck Depression Inventory II. Manual for the Beck Depression Inventory - II.

[86] Blevins CA, Weathers FW, Davis MT, Witte TK, Domino JL (2015). The Posttraumatic Stress Disorder Checklist for DSM-5 (PCL-5): Development and Initial Psychometric Evaluation. J Trauma Stress.

[87] Herdman M, Gudex C, Lloyd A, Janssen M, Kind P, Parkin D (2011). Development and preliminary testing of the new five-level version of EQ-5D (EQ-5D-5L). Qual Life Res.

[88] Guidance on reviewing and reporting unanticipated problems involving risks to subjects or others and adverse events. Office of Human Research Protections and the Department of Health and Human Services. January 15th, 2007.

[89] Chen C, Anderson K, Mehrotra DV, Rubin EH, Tse A (2018). A 2-in-1 adaptive phase 2/3 design for expedited oncology drug development. Contemp Clin Trials.

[90] Teasdale G, Jennett B (1974). Assessment of coma and impaired consciousness. A practical scale. Lancet.

[91] Chan AW, Tetzlaff JM, Altman DG, Laupacis A, Gotzsche PC, Krleza-Jeric K, et al. SPIRIT 2013 statement: defining standard protocol items for clinical trials. Ann Intern Med. 2013;158(3):200–207 10.7326/0003-4819-158-3-201302050-00583 PMC5114123 23295957

