# Supplementary material for: The Vitamin C, Thiamine and Steroids in Sepsis (VICTAS) Protocol: a prospective, multi-center, double-blind, adaptive sample size, randomized, placebo-controlled, clinical trial
Source: Trials. 2019 Apr 5;20:197. doi: 10.1186/s13063-019-3254-2 (PMC6451231; doi:10.1186/s13063-019-3254-2)
Supplement: Supplementary file 1 — Trial publication and data management protocol. (PDF 293 kb) [file 13063_2019_3254_MOESM1_ESM.pdf]

VICTAS™

Vitamin C, Thiamine and Steroids in Sepsis

Trial Publication and Data Management Protocol

Principal Investigators

Jon Sevransky, MD, MHS, FCCM
Emory University

Richard Rothman, MD, PhD
Johns Hopkins

David W. Wright, MD, FACEP
Emory University

Central IRB Approval:
Johns Hopkins Medicine IRB # 00164053

ClinicalTrials.gov Identifier:
NCT # 03509350

Study Sponsored by:
The Marcus Foundation

VICTAS Trial Procedures for the Development of Publications

Overview

The primary goal of the publications protocol is to ensure rapid and accurate translation of study results to the scientific, medical, and global community and maximize the scholarly productivity of the trial. This procedure protocol outlines the steps for publishing data related to the VICTAS trial, including concept generation and submission, writing and analysis responsibilities, and authorship. The publication process will be guided by the VICTAS publications committee (listed below). Writing groups will be created to analyze the data and prepare the publications.

The primary results generated from the study are the initial priority for publication. Following publication of the primary paper, several secondary hypotheses have been pre-identified that will be explored for publication. Additional ideas for manuscripts are highly encouraged. The VICTAS leadership considers it important to allow all interested investigators and VICTAS participants the opportunity to publish from the VICTAS data set. VICTAS affiliated personnel will have priority for manuscript development during the first year after the final analysis, however non-VICTAS parties can participate. Data will be released to the public per the pre-specified Data Sharing Plan.

VICTAS Publications Committee (VPC) Members

- Jon Sevransky - Pulmonary Critical Care, Emory (Chair)
- David W. Wright, Emergency Medicine Grady/Emory
- Tim Buchman - Surgery Critical Care, Emory
- Kate Heilpern - Emergency Medicine, Emory
- Craig Coopersmith - Surgery Critical Care, Emory
- Greg Martin - Pulmonary Critical Care, Grady/Emory
- Larry Busse - Pulmonary Critical Care, Emory University St. Joseph's
- Alex Hall - Emergency Medicine, Grady/Emory
- Caroline Rudolph - Surgery Critical Care, Emory
- Gabe Kelen - Emergency Medicine, Johns Hopkins
- Rich Rothman - Emergency Medicine, Johns Hopkins
- Dave Hager - Critical Care, Johns Hopkins
- Roy Brower - Critical Care, Johns Hopkins
- Jeremiah Hinson - Emergency Medicine, Johns Hopkins
- Alpha (Berry) Fowler - Critical Care, VCU
- Chris DeWilde - Critical Care, VCU
- Wes Ely - Critical Care, Vanderbilt
- Gordon Bernard - Critical Care, Vanderbilt
- Todd Rice - Critical Care, Vanderbilt
- Mark Levine, NIH
- Michael Hooper - Critical Care, East Virginia
- David Gaieski - Critical Care, Jefferson
- Roger Lewis – Berry Consulting, UCLA
- Top 3 Enrolling Site PIs

The VPC has the primary responsibility for overseeing submission of the VICTAS primary and secondary analyses, as pre-specified in the VICTAS Statistical Analysis Plan. The VPC will meet on an as needed basis

in response to requests and inquiries regarding VICTAS related publications. In addition, the VPC has the following responsibilities:

- Set publication priorities for the VICTAS trial data. Of note, no secondary analyses of the data may be submitted for publication prior to completion and acceptance of the primary manuscript.
- Review and approve all VICTAS publications (manuscripts, abstracts, posters, presentations) prior to the public release of data
- Facilitate writing groups and approve or assign a designated leader
- Ensure correct interpretation and representation of VICTAS data
- Ensure appropriate authorship designation and provide final ruling on disputes
- Track manuscript development and encourage timely submissions
- Evaluate outside requests for data use (prior to availability of public use data set)
- Approve the use of VICTAS data for grant submissions

General process for hypothesis / analysis submission and approval

- Submit idea / hypothesis via Web-based Portal (minimum 6 weeks prior to data need)
- Notify the Chair of the VPC of the submission via email, and alert to any time sensitive issues
    - VPC will then review the submission within 2 weeks
    - VPC will prioritize request and ensure no overlap with planned proposals
- Upon approval by the VPC, form Hypothesis Writing Group (HWG) with VPC's oversight
- Identify lead author/Chair of the writing group
- Submit detailed description of data items needed (per DCC requirements)
    - If DCC is providing statistical support, analysis will be coordinated with HWG
    - If DCC is not providing statistical support, a VICTAS Internal Trial Dataset (ITD) will be provided once available. This dataset is considered confidential and should only be used for the specific proposal at hand.
- Draft manuscript, create author list, and identify high impact journal for submission (draft should be in format per journal specifications – word counts, etc.)
- Present near-final draft to VPC
    - VPC reviews final manuscript
- After final approvals, ensure proper authorship and appropriate acknowledgement (see below) and submit final version to journal
- If significant rewrites or new analyses are required by the Journal, resubmission to VPC may be required

The VPC will attempt to engage and include as many interested investigators as possible. Site PIs, members of the CCC, and DCC have initial publication rights. Ideas not pre-specified in the primary and secondary analysis plan will be evaluated on a first-come, first-served basis. Once the hypothesis/manuscript idea has been vetted and approved by the VPC, an HWG will be formed from the interested parties. The HWG will propose a Group leader, who will serve as the organizer, delegate and direct the manuscript writing process, oversee a timely draft submission, and ensure the highest quality manuscript possible. The final draft must be approved by the VPC before submission to a journal. The VPC retains the right (and ultimate authority) to appoint members to the writing group, assign a group leader, disapprove a manuscript, require edits to the manuscript, and/or reassign a Group leader (e.g poor progress, significant dissention, other).

Primary Hypothesis/Manuscript
The VPC will serve as the writing group for the manuscript reporting the primary outcome and select supporting secondary analyses for the VICTAS trial. The VICTAS Statistical Analysis Plan specifies the analyses that will be conducted and presented in the primary paper. The VICTAS PI will Chair the writing group and develop the initial draft. Final authorship will be guided by the International Committee of Medical Journal Editors' rules for authorship and will include a list of key contributors and the statement referencing the supporting participants: *"on behalf of the VICTAS Investigators"*. The full list of VICTAS investigators will be listed per the journal's specifications (appendix, supplement, etc.) with the intention of authorship acknowledgement and contribution. The primary paper should be completed within 6 months of the primary data analysis availability. The Primary data will be published regardless of the study results.

Secondary Manuscripts - Preplanned Hypotheses
In addition to the primary hypothesis, several secondary hypotheses were pre-identified for analysis and potential publication. The VPC will work with VICTAS investigators and team members to garner levels of interest and form writing groups. HWGs will include individuals with expertise in the area of interest (e.g. Vitamin C / biomarker writing group), and other skills (e.g. statistics). The Group leader, most often the primary author, will organize the HWG, direct hypothesis development, and oversee the writing of the publication. Group leaders will also determine authorship per the authorship guidelines outlined in this document (Authorship) and ensure expeditious high quality manuscript submission.

Tertiary Manuscripts
Tertiary papers are post-hoc analyses that relate to the central hypotheses being tested, but not pre-specified in the SAP. HWGs will be formed as described for the secondary manuscripts.

Quaternary and Newly Generated Hypotheses
Quaternary papers utilize the dataset for data that most often do not relate to the initial hypotheses of the study. The VICTAS trial was a rigorously conducted Phase III clinical trial that includes a treasure trove of information and data from multiple sources (e.g. epidemiology, clinical assessments, clinical management, vital signs, laboratory data, confounding conditions, surgical interventions, adverse events information, and a multitude of outcome measures). It is anticipated that numerous new hypotheses will be generated to advance the knowledge of sepsis patients. The VICTAS PI and or designees have the first rights to publish collective study data per the VPC approval. VICTAS team members are highly encouraged to review the case report forms and consider important clinical questions that can be answered with the VICTAS data. The process for submitting and authorship will follow the standard guidelines in this document.

Methods papers and Study-independent Publication Procedures
Members of the Executive committee, CCC, DCC and Site PIs may wish to publish methods papers that describe the VICTAS operations, network's function, or papers that are otherwise wholly independent from the trial conducted. These paper proposals and final manuscripts will be submitted to the VICTAS VPC.

Abstracts, Posters and Oral Presentations
Publications and presentations of all types that use VICTAS data require prior approval, and in general will follow the same process as for manuscripts. Conversion of abstracts, posters and oral presentations into full manuscripts is highly encouraged and in most cases mandatory. After acceptance to the presentation forum (scientific meeting, assembly, other), a draft manuscript should be submitted to the VPC prior to

the actual meeting presentation.

Acknowledgements

Acknowledgements at a minimum should include the following statement: *Research reported in this publication was supported by the Marcus Foundation, and coordinated through Emory University, Grady Memorial Hospital, Johns Hopkins University, and Vanderbilt University Medical Center (VUMC). The content is solely the responsibility of the authors and does not necessarily represent the official views of the Foundation or other supporting entities*. Other acknowledgements will depend on the specific manuscript.

IRB and Clinical Trials.gov

The publication should also include the statement that the research project was:

- approved locally by each site through the Johns Hopkins University single Institutional Review board process.
- submitted to Clinicaltrial.gov

Authorship

Authorship on VICTAS related publications will be guided by the International Committee of Medical Journal Editors (ICMJE) recommendations. The ICMJE recommends that authorship be based on the following 4 criteria (quoted from the ICMJE website):

- Substantial contributions to the conception or design of the work; or the acquisition, analysis, or interpretation of data for the work; AND
- Drafting the work or revising it critically for important intellectual content; AND
- Final approval of the version to be published; AND
- Agreement to be accountable for all aspects of the work in ensuring that questions related to the accuracy or integrity of any part of the work are appropriately investigated and resolved.

Group authorship is encouraged. In general, all members of the VPC and the HWG that significantly contribute to the content of the manuscript should be included. VICTAS publications will include the phrase "*…for the VICTAS Trial Investigators*" at the end of the author list. All VICTAS Trial Investigators and coordinators will be named in the publication in a format that meets the journal of submission's guidelines. Additionally the VICTAS PIs (usually listed as the last 3 names in the formal list), CCC PI, DCC PI and manuscript statistician will also be included as authors and be required to participate as authors based on the guidelines for authorship. Order of authors between the first author and last author will be the responsibility of the lead author and consensus of the HWG. Group leaders should attempt to resolve any authorship issues or disputes early in the writing process. The VPC/VICTAS PI will have final word on authorship in settings where resolution cannot be reached within the HWG.

The authors of the publication are solely responsible for the manuscripts' content. Review and/or edits made at the request of an outside organization, individuals, funder, or supplier will be considered by the VPC but not required for publication.

Approval Process and Expected Timelines

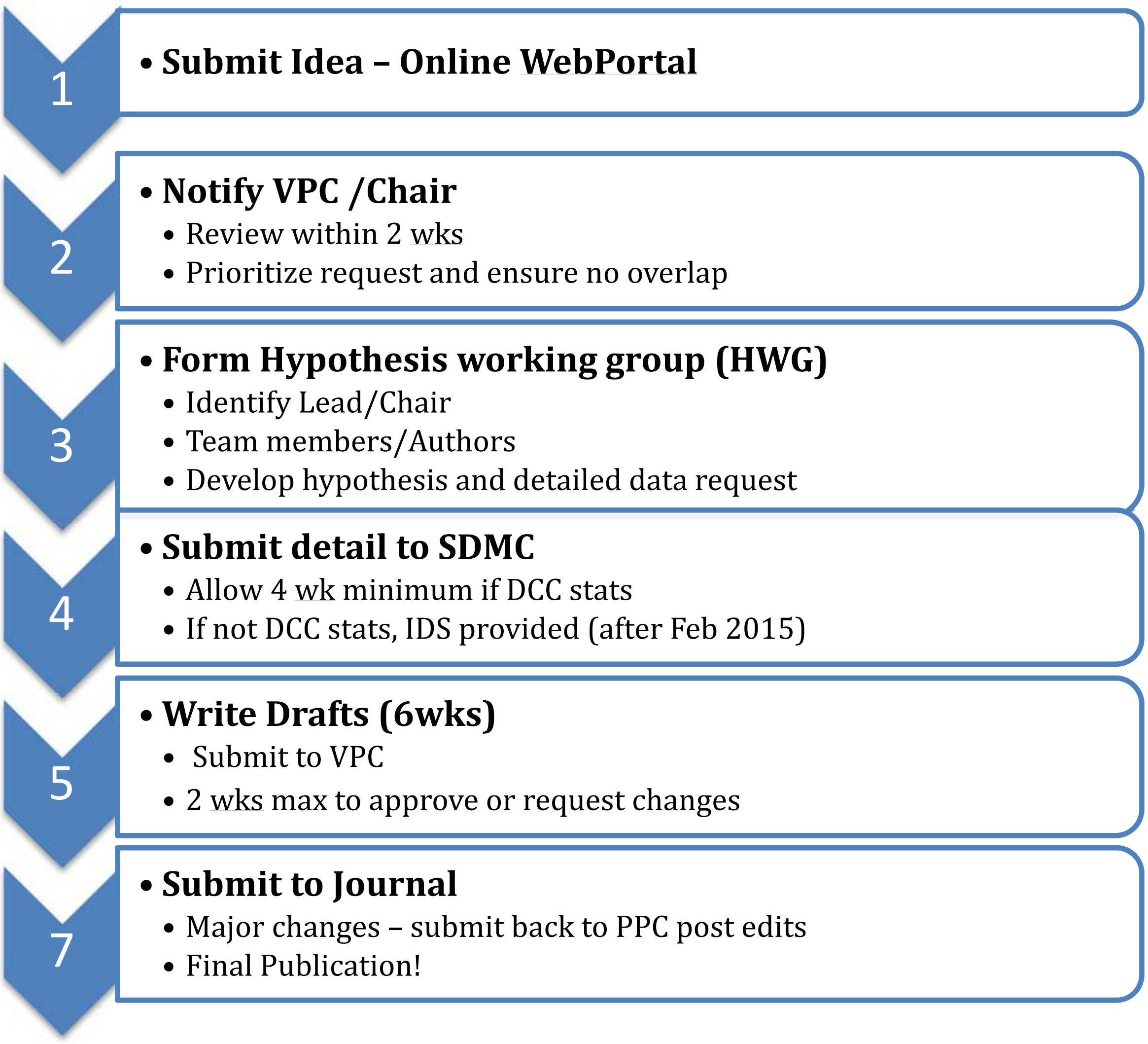

Typical Timelines:
Submit Idea to Web portal and notify VICTAS Publications Committee (VPC) ---> Review by VPC (2 wks) ---> OK'd – set up HWG, provide detail data/hypotheses request to VICTAS Vanderbilt Data Coordinating Center (VVDCC) (variable time) ---> VVDCC to provide data, database, or analysis depending on agreement (4 wks minimum) ---> Manuscript draft by HWG (6 wks) ---> Submit semifinal draft to VPC (2 wks) ---> Edit or ok to submit to Journal (2 wks) ---> Journal review (variable) ---> Edit – resubmit to VPC if major (2 wks) ---> Final Submit to Journal.

The purpose of the approval process is to help track and *prioritize* publications, prevent overlap of ideas and data analysis, ensure fair acknowledgement of the idea's originator(s) and authors, assist with HWG development, and ensure the highest quality and timely publications. In addition, significant resources are often required to prepare and provide the variety of datasets needed for hypothesis testing and analysis.

At least 4 weeks are required to obtain data from DCC, with more time allowed for specific analyses. Respect of the DCC's time and role in this process is important and will be monitored on an ongoing basis. Requests for urgent reviews/analyses will go to the VPC and accommodated if possible, but cannot be

guaranteed.

Confidentiality and Data Release

No VICTAS data should be released to the public or non-VICTAS entities unless prior approval is obtained from the VPC or VICTAS Executive committee. All data and correspondence regarding publications are considered confidential until approval for release. All data associated with VICTAS, whether locally or centrally stored should be de-identified prior to release, sharing, or publication. Investigators are not allowed to present data gathered from their clinical sites before the primary analyses are published. VICTAS results should not be discussed with the media without authorization from the VICTAS PI.

Only the trial PIs and their designees, Site PIs, and members of the CCC and DCC have collective data rights until one year after the publication of the primary data. Individual Institutions shall retain ownership of all data that they generate. Institutions shall grant to VICTAS non-exclusive license to use data for educational and research purposes. The VPC will retain oversight of the collective data and decision-making authority with respect to the collective data prior to public release. Finally, one year following the publication of the primary results, the public use data set will be created for public use. Clinical data, biomarker data, and other data will be linked through a pseudo Global Uniform Identification number (Pseudo-GUID). Date and/or time shift procedures may be utilized if the VICTAS Executive Committee and DCC determine that this additional layer of de-identification is required prior to release of the public use data set.

Individual SITE Investigator Publication Rights

Site Investigators who wish to publish their own institution's data will be able to proceed with such publication, provided that the Site PI has first sought approval for multi-site publication in accordance with the procedures set out under the study-specific publication procedure.

Adherence to Policy

Participation in VICTAS requires adherence to the publication policy described in this SOP, even though Site PIs retain ownership of the data collected at their sites. Authors who publish articles that are not compliant with this policy must contact the journal and retract the publication.

Journal Submission

Within 2 months of receiving the Publications Committee comments and approval, the revised (if necessary) manuscript will be circulated by the HWG Chair to the other members of the HWG for final sign-off. The HWG members have no more than 14 days to review the final manuscript. A copy of the journal cover letter and final draft of the manuscript must be sent to the Publications Committee in addition to all co-authors.

The HWG Chair must keep the VPC and the co-authors informed as to the manuscript's progress through the journal review process. Following the final acceptance by the journal, the HWG Chair is responsible for providing the Chair of the VPC with the manuscript and all graphics and supplemental materials associated with the manuscript. Upon publication of the manuscript, the HWG Chair must provide either a reprint or copies of the final publication, and all graphics and supplemental materials associated with the manuscript, to the VPC.

If there are substantive changes made in the manuscript during the journal review process (major findings or conclusions, alterations of the sample, exclusion/inclusion of major covariates), the revised manuscript should be submitted to the VPC for re-review prior to resubmission to the journal. The revised manuscript must be resubmitted within 3 months of receiving the journal reviews of the original submission.

Study Data

Emory, VUMC, and Johns Hopkins University shall share and have all rights to data and results generated in the conduct of the Study ("Study Data"). The Executive committee, also serving as the publications committee, shall be afforded an opportunity to utilize the Study Data as first right of publication, except that any use or disclosure of Protected Health Information, as defined in the Health Insurance Portability and Accountability Act of 1996 and its implementing regulations ("HIPAA"), shall be subject to the authorization provided by the study subject in the informed consent or other authorization document and applicable laws. Notwithstanding the foregoing, no entity shall use any Study Data in filing and/or support of a patent application, without approval from Emory.

Data Use/Ownership "Data" shall mean all data and information generated by Institution as a result of conducting the Study in accordance with the IRB approved Protocol. Data does not include original Study subject or patient medical records, research notebooks, source documents, or other routine internal documents kept in the Institution's ordinary course of business operations, which shall remain the sole and exclusive property of the Institution or medical provider. Study leadership from Emory University, Vanderbilt University, and Johns Hopkins University shall own and have the right to use the Data in accordance with the signed informed consent and authorization form, applicable laws, and the terms of this Agreement. Notwithstanding any licenses or other rights granted to Sponsor herein, but in accordance with the confidentiality and publication sections herein, Institution shall retain the right to use the Data and results for its publication, IRB, regulatory, legal, clinical, educational, and internal research purposes.

Access to Records

VUMC agrees to maintain and preserve the documents, original records and Study Data that result from the Study in accordance with applicable laws.

Data Sharing Plan for VICTAS

The VICTAS trial sites will share all data with the VICTAS Johns Hopkins Coordinating Center (VJHCC), the VICTAS Vanderbilt Data Coordinating Center (VV) and Emory University. The VICTAS philosophy includes making de-identified data available to both scientists and the general community of clinicians that will use their results to drive patient care.

Our plan includes:

- Presenting at national scientific meetings and publishing articles in scientific publications.
- Advocacy and outreach efforts performed and studied by the Clinical Translation Unit.
- Creation and maintenance of a data enclave by the VVDCC; a controlled, secure environment in which eligible researchers can perform analyses using de-identified data sets.

After completion of the trials *and* dissemination of primary study results, the analysis data files will be made available to the public, along with the final version of the study protocols, the data dictionary, and a brief instruction. VICTAS investigators will have exclusive opportunities to digest the study results and generate further hypotheses, and submit manuscript proposals, for a period of 1 year prior to public data release. The rationale for the timeline is to ensure that priority is given to the VICTAS investigators in the analysis of the data. The study datasets generated from VVDCC database will subscribe to the common data elements as much as possible.

De-identification of Shared Data

In compliance with the HIPAA regulations, the analysis data files that will be made publicly available will undergo the following de-identification process:

- delete study ID numbers and assign a random number to each subject
- delete site ID numbers and assign a random number to each clinical site
- delete investigator or assessor name/ID
- delete the randomization date but keep the month and year and add the order in which patients enrolled
- convert all dates and times (e.g., birthdate, death date, end of study date) to the number of days/minutes from the date and time of randomization.

Resource and Biologicals Sharing

Biological (serum, blood samples) sharing will be covered in a separate SOP.
