# Supplementary material for: The Vitamin C, Thiamine and Steroids in Sepsis (VICTAS) Protocol: a prospective, multi-center, double-blind, adaptive sample size, randomized, placebo-controlled, clinical trial
Source: Trials. 2019 Apr 5;20:197. doi: 10.1186/s13063-019-3254-2 (PMC6451231; doi:10.1186/s13063-019-3254-2)
Supplement: Supplementary file 2 — Adaptive design report. (PDF 3433 kb) [file 13063_2019_3254_MOESM2_ESM.pdf]

Adaptive Design Report for a Trial of the Virginia Cocktail

Submitted to VICTAS

April 26, 2018

1 Introduction

This document describes the adaptive design for a randomized clinical trial to investigate the efficacy of the combined use of vitamin C, corticosteroids and thiamine (hereafter "Virginia Cocktail") for patients with sepsis. The trial employs a novel endpoint that formally incorporates a patient's risk of death as well as the amount of time spent on pressors or mechanical ventilators. This second component of the endpoint may be thought of as the "survivor benefit", which captures a patient's speed of recovery. Mortality rate is a key secondary endpoint for the trial.

The trial has a flexible sample size that will be determined adaptively using a "Goldilocks" strategy. The trial will enroll up to 2000 subjects. Accrual may stop after 200, 300, 400, 500, 1000, 1500, or 2000 subjects, randomized equally between Placebo and the Virginia Cocktail. The first three interims at 200, 300, and 400 may stop accrual for evidence of an overwhelming benefit on the mortality endpoint. However, if the data at these early interims are indeterminate on mortality, the trial may continue to a larger sample size (up to $N = 2000$) with the primary endpoint of vasopressor and ventilator-free days. When the data are sufficiently strong at these additional interims (either positive or negative) on the primary endpoint, the trial may select a sample size below the maximum, but when necessary (e.g. for a moderate effect), the trial may continue to the larger sample size.

Due to the importance of the secondary mortality endpoint, the trial also allows for "bypassing" an early stop on the primary endpoint if there is sufficiently high chance remaining that the mortality test would be successful with continued enrollment.

The overall type I error rate for the trial is controlled at one-sided 2.5%. The early interims have conservative rules for spending alpha so that 0.1% will be used up to $N = 400$ and the remaining 2.4% is reserved for $N = 500$ or beyond.

This adaptive design report fully describes the decision rules for the adaptive design, including the statistical models, and provides operating characteristics of the design determined through clinical trial simulation.

1.1 Treatment Arms

Up to 2000 subjects will be randomized in a 1:1 ratio between two arms:

- Control: Placebo
- Treatment: Virginia Cocktail (VC), which is the combined use of Vitamin C, steroid, and thiamine

1.2 Endpoints

Vasopressor and Ventilator-free days (VVFD): The primary endpoint for this trial is the number of consecutive days free of pressors and mechanical ventilation in the first 30 days after start of treatment. The endpoint will be recorded to the nearest day. Patients who die are scored zero VVFD, and patients who must return to ventilation and/or vasopressors will have their counters reset at zero days.

There are two mechanisms by which the endpoint VVFDs may be reduced relative to Placebo. First, the Virginia Cocktail may reduce the risk of death. Second, the Virginia Cocktail may reduce the number of days spent on ventilation for those subjects that do not die. Thus there may be benefit in mortality, in speed of recovery, or both -- each translating to an improvement in VVFD.

Mortality: A key secondary aim of the trial is to assess the potential benefit of the Virginia Cocktail on the 30-day mortality rate. However, as the required sample size for showing mortality benefit is quite large, the trial is likely to be underpowered for this endpoint.

2 Adaptive Design

2.1 Final Analysis

The final analysis will occur after all randomized subjects have been followed to their primary endpoint.

The final analysis of the VVFD endpoint is a Wilcoxon rank-sum test (also known as Mann-Whitney U test) which will be tested using one-sided alpha of 0.022. In the final analysis, subjects who died are treated in the same way as subjects who survived but had zero VVFD.

The secondary goal of the trial is to demonstrate superiority of the Virginia Cocktail relative to Placebo in reducing the mortality rate:

$$H_0: q_{VC} = q_{PBO} \qquad \text{vs.} \qquad H_A: q_{VC} < q_{PBO}.$$

Here q_j is the mortality rate for arm j ($j = PBO$ for Placebo, $j = VC$ for the Virginia Cocktail). The mortality endpoint will be tested using a Chi-square test.

The type I error rate will be controlled by employing a gatekeeping strategy. Thus testing will take place in a hierarchical manner so that:

1. If the selected sample size is $N < 500$, the mortality endpoint is tested first using one-sided alpha 0.001. The VVFD endpoint will only be tested if the mortality endpoint is successful. If so, VVFD will be tested with one-sided alpha 0.022.
2. If the selected sample size is $N \geq 500$, the VVFD endpoint is tested first using one-sided alpha 0.022. The mortality endpoint will only be tested if the VVFD endpoint meets success. If so, mortality will be tested with one-sided alpha 0.024.

These thresholds have been adjusted to control the overall type I error rate of the trial at 2.5%, accounting for the possibility of stopping accrual early.

2.2 Interim Analyses for Sample Size Selection

2.2.1 Interim Timing

2.2.1.1 Early Interims for Mortality

Early interim analyses focused on detection of a large mortality effect will be conducted at 200, 300 and 400 subjects randomized. At each of these points, the predictive probability of achieving significance on mortality (with one-sided alpha 0.001) with the current sample size will be computed. If the predictive probability exceeds 90%, study accrual will be stopped. All currently randomized subjects will continue follow up and the final analysis will be performed after all currently randomized subjects have been followed to their final outcome. In this case, the primary analysis will be performed on mortality. The test on VVFD will only be performed if statistical significance is met on mortality.

2.2.1.2 Additional Interims

If the conditions for stopping accrual with $N < 500$ for mortality benefit are not met, the trial will continue enrollment with VVFD as the primary endpoint. These additional interim analyses will be conducted every 500 subjects randomized. At each interim, the following actions may result:

1. Stop the trial for futility on VVFD
2. Stop accrual for expected success on both VVFD and mortality endpoints
3. Stop accrual for expected success on VVFD alone
4. Continue to the next analysis.

Each of these possible actions will be described in greater detail below.

2.2.2 Predictive Probabilities

At each interim analysis, we compute two predictive probabilities for the VVFD endpoint for use in interim decisions:

- PP_{VVFD} (current N): the predictive probability of success on the primary VVFD endpoint if enrollment stops with the current sample size, and all currently enrolled subjects are followed to their primary endpoint.
- PP_{VVFD} (max N): the predictive probability of success on the primary VVFD endpoint if enrollment continues to the maximum number of subjects.

Similarly, we compute two predictive probabilities for the morality endpoint:

- PP_{mort} (current N): the predictive probability of success on the mortality endpoint if enrollment stops with the current sample size, and all currently enrolled subjects are followed to their primary endpoint.
- PP_{mort} (max N): the predictive probability of success on the mortality endpoint if enrollment continues to the maximum number of subjects.

Statistical details relating to the computation of these predictive probabilities may be found in the Appendix.

2.2.3 Futility

Starting at $N = 500$, the trial will stop for futility if the predictive probability of eventual success on the VVFD endpoint at the maximumum sample size is less than 10%. That is, stop for futility if

$$PP_{VVFD}(\max N) < 0.10.$$

Thus we stop the trial for futility if there is little chance of ever detecting a statistically significant benefit on the primary VVFD endpoint, even with the maximum resource expenditure.

2.2.4 Stop Accrual for Expected Success on Both Endpoints

Accrual to the trial may stop for expected success on both endpoints if the predictive probability of eventual success with the current sample size exceeds 95% for both VVFD and for mortality. That is, stop accrual for expected success if

$$PP_{\text{VVFD}}(\text{current } N) > 0.95 \qquad \text{AND} \qquad PP_{\text{mort}}(\text{current } N) > 0.95.$$

Thus if both endpoints are sufficiently promising with the current sample size, enrollment will stop. In order to meet these thresholds, the data must already be strongly positive, and furthermore, there must be little risk that the subjects with outstanding data might reverse the success once all data become available.

If these thresholds are reached, then no additional subjects will be enrolled. Follow-up of currently enrolled subjects will continue and the final analysis will be

performed after all currently enrolled subjects are followed to their final 30-day outcomes.

2.2.5 Stop Accrual for Expected Sucess on VVFD Alone

Accrual to the trial may also stop for expected success on the primary endpoint of VVFD alone. This requires that the predictive probability of detecting a statistically significant beneficial effect on VVFD at the current sample size exceeds 95% and that the predictive probability of achieving a statistically significant benefit on mortality at the maximum sample size is less than 10%. Thus, if the trial is highly likely to be successful on VVFD with the current sample size, but mortality is unlikely to be successful even with continued enrollment, then accrual will stop:

$$PP_{\text{VVFD}}(\text{current } N) > 0.95 \qquad \text{AND} \qquad PP_{\text{mort}}(\max N) < 0.10.$$

Under these circumstances, success on mortality is likely out of reach, so the trial will conclude enrollment. This essentially acts as a futility rule on the mortality endpoint. Note that if the predictive probability for VVFD meets the stopping threshold, but the predictive probability for mortality exceeds 10%, then enrollment will continue, effectively "bypassing" the early success on VVFD in the hope of picking up success on the mortality endpoint. This bypass rule will be discussed further in the next section.

The final analysis will occur after all enrolled subjects have completed the 30-day follow up.

2.2.6 Continue to the Next Analysis

If no condition for stopping accrual is met at the interim, then the trial will continue accrual to the next analysis time (either the next scheduled interim, or to full enrollment and final analysis). Accrual to the trial does not stop during the interim.

There are situations for which accrual continues after VVFD meets the criteria to stop accrual for expected success but mortality does not. Mortality is a key secondary endpoint for this trial. Thus, if the trial meets the condition to stop accrual for expected success on VVFD, and mortality has a chance of being successful with additional sample size, there may be value to continuing enrollment to a larger sample size (thus "bypassing" the early success on VVFD alone). In summary, we will bypass a likely VVFD success if BOTH:

1. it is unlikely that mortality will be successful with the current sample size, and
2. we are hopeful we might win mortality later.

2.3 Summary of Interim Decision Rules

The table below summarizes the rules for stopping accrual.

Table 1: *Interim decision rules*

Sample Size	Interim Decision	Condition for Decision
N < 500	Futility	may be recommended by DSMB
	Expected success (mortality)	PP_{mort} (current N) > 0.90
	Continue	PP_{mort} (current N) < 0.90
N ≥ 500	Futility	PP_{VVFD} (max N) < 0.10
	Expected success (both endpoints)	PP_{VVFD} (current N) > 0.95 AND PP_{mort} (current N) > 0.95
	Bypass	PP_{VVFD} (current N) > 0.95 AND PP_{mort} (max N) > 0.10
	Expected success (VVFD only)	PP_{VVFD} (current N) > 0.95 AND PP_{mort} (max N) < 0.10
	Continue	PP_{VVFD} (max N) > 0.10 AND PP_{VVFD} (current N) < 0.95

3 Example Trials

In this section, we present two example trials to illustrate the adaptive process. For the selected trials, each analysis is represented by a series of tables.

3.1 Example Trial 1

Figure 1 shows the outcome of each analysis for one example simulated trial. The first interim occurs after 200 subjects randomized, 100 per arm. At the time of the interim, there are 85 total subjects whose final outcome is unknown (43 on Placebo, and 42 on the Virginia Cocktail). On the primary endpoint, the mean VVFDs is 16.1 days on the Placebo arm (with standard deviation 8.7) versus 20.1 days on the Virginia Cocktail (with standard deviation 6.1).

	N = 200		N = 300		N = 400		Final	
	Placebo	Cocktail	Placebo	Cocktail	Placebo	Cocktail	Placebo	Cocktail
Num Enrolled	100	100	150	150	200	200	200	200
Num Unknown	43	42	38	39	42	43	0	0
VVFD								
Observed [Mean (sd)]	16.1 (8.7)	20.1 (6.1)	16.2 (9)	19.9 (6)	16.4 (9)	19.8 (5.6)	16.2 (9)	19.8 (5.9)
p–value		0.0051		0.0032		0.0059		<0.001
Pred. Prob. current N		0.98		0.994		0.988		
Pred. Prob. max N		>0.999		>0.999		>0.999		
Mortality								
Observed [x/n]	10/57	3/58	22/112	6/111	30/158	7/157	39/200	10/200
Observed [%]	17.5%	5.2%	19.6%	5.4%	19%	4.5%	19.5%	5%
p–value		0.0181		<0.001		<0.001		<0.001
Pred. Prob. current N		0.329		0.862		0.998		
Pred. Prob. max N		0.955		0.998		>0.999		

Figure 1: *Example Trial 1*

The improvement in VVFD is bolstered by the large difference in mortality rates between arms. The observed rate on the Placebo arm is 17.5% (10.57) while the rate on the Virginia Cocktail is 5.2% (3/58). With 85 subjects data outstanding, the predictive probability of success on mortality with the current sample size is 0.329. Thus the trial continues to the next interim.

The second interim is performed after the 300th subject randomized. The observed mortality rates are 19.6% and 5.4%. The trial is likely to be successful (predictive probability 0.862), but there is still enough uncertainty in the outstanding data, that the trial has not reached the threshold to stop for expected success.

At the third interim, there are 200 subjects enrolled per arm and a total of 315 subjects whose 30-day outcome is known. The difference in observed mortality rates is 14.5% (19% on Placebo versus 4.5% on Virginia Cocktail). The trial is highly likely to remain successful after follow up of the remaining subjects. Thus the trial stops accrual with a sample size of $N = 400$. The final analysis occurs once all subjects have been followed to completion. At the final analysis, the mortality rates are 19.5% versus 5%, resulting in a p-value of < 0.001. Thus the trial is successful on the mortality endpoint, and goes on to test the VVFD endpoint. The p-value for VVFD is also < 0.001, so the trial also meets the success boundary for VVFD.

3.2 Example Trial 2

In Example trial 2, the study goes through the early interims without stopping early because the predictive probability of success with the current sample size on the mortality endpoint never exceeds 90%. These early interims are shown in Figure 2.

	N = 200		N = 300		N = 400	
	Placebo	Cocktail	Placebo	Cocktail	Placebo	Cocktail
Num Enrolled	100	100	150	150	200	200
Num Unknown	38	37	41	41	37	36
VVFD						
Observed [Mean (sd)]	16.1 (8.9)	14.9 (9.7)	14.8 (9.5)	16 (9.4)	14.1 (9.9)	16.3 (9.6)
p–value		0.7339		0.1727		0.0106
Pred. Prob. current N		0		0.104		0.896
Pred. Prob. max N		0.358		0.798		0.95
Mortality						
Observed [x/n]	13/62	16/63	29/109	24/109	50/163	37/164
Observed [%]	21%	25.4%	26.6%	22%	30.7%	22.6%
p–value		0.7212		0.2149		0.0484
Pred. Prob. current N		0		0		0.002
Pred. Prob. max N		0.147		0.572		0.828

Figure 2: *Example Trial 2, early interims*

Figure 3 shows the additional interims for this example. After 500 subjects enrolled, the Placebo arm has mean VVFD 14.5 days versus 16.5 days on the Virginia Cocktail, and the predictive probability of success with the current sample size is 97.6%,

greater than the 95% threshold for stopping accrual. Looking at the mortality endpoint, the Virginia Cocktail reduces the rate to 21.8% from the Placebo rate of 28%. This endpoint is unlikely to be successful with the current sample size (16.2% predictive probability), but has greater than 10% chance (in fact, 74.5% chance) of being successful at a later time. This circumstance results in a bypass of the early stop for VVFD. Enrollment continues to the next interim.

	N = 500		N = 1000		N = 1500		Final	
	Placebo	Cocktail	Placebo	Cocktail	Placebo	Cocktail	Placebo	Cocktail
Num Enrolled	250	250	500	500	750	750	750	750
Num Unknown	39	39	35	35	34	34	0	0
VVFD								
Observed [Mean (sd)]	14.5 (9.6)	16.5 (9.5)	14.5 (9.6)	16.3 (9.4)	14.6 (9.5)	16.3 (9.3)	14.7 (9.5)	16.2 (9.4)
p–value		0.0034		<0.001		<0.001		<0.001
Pred. Prob. current N		0.976		>0.999		>0.999		
Pred. Prob. max N		0.986		>0.999		>0.999		
Mortality								
Observed [x/n]	59/211	46/211	130/465	104/465	192/716	154/716	197/750	164/750
Observed [%]	28%	21.8%	28%	22.4%	26.8%	21.5%	26.3%	21.9%
p–value		0.0716		0.0247		0.0095		0.0231
Pred. Prob. current N		0.162		0.56		0.982		
Pred. Prob. max N		0.745		0.796		0.896		

Figure 3: *Example Trial 2, additional interims*

At the $N = 1000$ interim, the trial maintains the high predictive probability of success on VVFD. The mortality endpoint still has insufficiently high predictive probability of success with the current sample size, but it is still likely (79.6%) that success may eventually be attained. Again, the trial bypasses the VVFD success and continues accrual.

By the $N = 1500$ analysis, the mortality endpoint now also has greater than 95% predictive probability of success. Thus, the trial stops accrual with the currently enrolled 1500 subjects. The final analysis occurs after all subjects have been followed to completion. At the final analysis, the Wilcoxon p-value for VVFD is < 0.001 (below the 0.022 threshold), resulting in primary endpoint success. The mortality endpoint has p-value 0.0231 < 0.024, resulting in a success on the secondary endpoint.

In summary, this trial bypassed an early stop on VVFD, adding an additional 1000 subjects before stopping accrual for expected success on both endpoints. At the final analysis, the trial was successful on both endpoints.

3.3 Example Trial 3

Example trial 3 begins by randomizing 100 subjects to each arm (Figure 4). At each of the early interims at 200, 300, and 400 subjects randomized, the trial does not meet the criterion to stop for expected success on mortality. At the time of the

$N = 500$ interim analysis (Figure 5), 75 subjects have unknown outcomes. The observed VVFD means are differ by 1 day, resulting in about 36% predictive probability of success with the current sample size. The predictive probability of success at the maximum sample size is quite high at 91.8%, exceeding the 10% futility boundary, so accrual continues to 1000 subjects.

	N = 200		N = 300		N = 400	
	Placebo	Cocktail	Placebo	Cocktail	Placebo	Cocktail
Num Enrolled	100	100	150	150	200	200
Num Unknown	38	38	38	37	39	39
VVFD						
Observed [Mean (sd)]	15.1 (9)	15.9 (8.4)	15.6 (8.7)	16 (9)	15.9 (8.8)	16.6 (9.1)
p–value		0.3031		0.2648		0.1109
Pred. Prob. current N		0.068		0.024		0.104
Pred. Prob. max N		0.672		0.706		0.734
Mortality						
Observed [x/n]	14/62	11/62	23/112	23/113	33/161	32/161
Observed [%]	22.6%	17.7%	20.5%	20.4%	20.5%	19.9%
p–value		0.251		0.4865		0.4448
Pred. Prob. current N		0.001		0		0
Pred. Prob. max N		0.576		0.245		0.223

Figure 4: *Example Trial 3, early interims*

By the next interim, the arms still show about 1 day of separation in mean VVFD, but the additional data has reduced variability around these estimates, and the predictive probability of success with the current sample size exceeds 95%. Turning to the mortality endpoint, there is very little difference between the arms, so that the probability of winning mortality with the current sample size is essentially zero. There is also very low chance that additional enrollment will result in a successful outcome on mortality, with predictive probability of only 5.7%. Thus the trial stops accrual, expecting success only on the VVFD endpoint.

	N = 500		N = 1000		Final	
	Placebo	Cocktail	Placebo	Cocktail	Placebo	Cocktail
Num Enrolled	250	250	500	500	500	500
Num Unknown	38	37	39	39	0	0
VVFD						
Observed [Mean (sd)]	15.7 (9.1)	16.7 (9.1)	15.6 (9.1)	16.6 (9.4)	15.7 (9.1)	16.6 (9.4)
p–value		0.051		0.0066		0.0064
Pred. Prob. current N		0.358		0.976		
Pred. Prob. max N		0.918		0.974		
Mortality						
Observed [x/n]	46/212	42/213	100/461	98/461	108/500	106/500
Observed [%]	21.7%	19.7%	21.7%	21.3%	21.6%	21.2%
p–value		0.3073		0.4363		0.4387
Pred. Prob. current N		0		0		
Pred. Prob. max N		0.328		0.057		

Figure 5: *Example Trial 3, additional interims*

After full followup of the 1000 enrolled subjects, the Wilcoxon p-value for VVFD is 0.0064, resulting in success for VVFD. The mortality p-value is 0.4387.

3.4 Example Trial 4

In this trial, accrual does not stop at the early interims (Figure 6). At the $N = 1000$ interim (Figure 7), the observed mean VVFD is very similar between the two arms, with virtually no chance that the subjects with outstanding could be sufficient to conclude success. There is a 9.4% predictive probability that future data could result in success, and the trial stops for futility.

	N = 200		N = 300		N = 400	
	Placebo	Cocktail	Placebo	Cocktail	Placebo	Cocktail
Num Enrolled	100	100	150	150	200	200
Num Unknown	43	44	40	39	38	38
VVFD						
Observed [Mean (sd)]	14.6 (9.7)	16.6 (9.2)	13.3 (10.2)	15.9 (8.9)	13.8 (9.9)	15 (9.5)
p–value		0.1539		0.069		0.1859
Pred. Prob. current N		0.35		0.492		0.034
Pred. Prob. max N		0.884		0.968		0.696
Mortality						
Observed [x/n]	13/57	11/56	35/110	22/111	48/162	40/162
Observed [%]	22.8%	19.6%	31.8%	19.8%	29.6%	24.7%
p–value		0.3405		0.0207		0.1588
Pred. Prob. current N		0.002		0.107		0
Pred. Prob. max N		0.468		0.931		0.591

Figure 6: *Example Trial 4, early interims*

	N = 500		N = 1000		Final	
	Placebo	Cocktail	Placebo	Cocktail	Placebo	Cocktail
Num Enrolled	250	250	500	500	500	500
Num Unknown	42	42	41	41	0	0
VVFD						
Observed [Mean (sd)]	14.1 (9.6)	15 (9.4)	14.9 (9.5)	15.2 (9.3)	14.7 (9.5)	15 (9.4)
p–value		0.205		0.4207		0.3427
Pred. Prob. current N		0.004		0		
Pred. Prob. max N		0.592		0.076		
Mortality						
Observed [x/n]	56/208	50/208	114/459	109/459	128/500	122/500
Observed [%]	26.9%	24%	24.8%	23.7%	25.6%	24.4%
p–value		0.2498		0.3502		0.3306
Pred. Prob. current N		0.002		0		
Pred. Prob. max N		0.401		0.094		

Figure 7: *Example Trial 4, additional interims*

4 Simulation Scenarios

The operating characteristics of this trial were determined through trial simulation. We hypothesized several scenarios for the underlying treatment effect on mortality and speed of recovery, and simulated the entire trial multiple times under each scenario. In each virtual trial, the interim analyses were conducted according to the pre-specified rules, and results were tracked for each trial, including whether the trial was successful, the selected sample size, etc. This section describes the algorithm and the parameters that were used to simulate subject-level data for the virtual trials. These assumptions are used only in the generation of virtual subjects, and are not part of the analysis of subject data (virtual or real).

4.1 Virtual Subject Response Profiles

In order to simulate the outcome of a virtual subject, we first simulate whether the subject dies (hence VVFDs will be 0). If the simulated subject does not die, we then simulate the number of days free of pressors and ventilators for that subject.

4.1.1 Mortality Rate Profiles

As a default scenario, we simulate a Placebo subject as having 25% probability of death. Scenarios for the mortality reduction in the Virginia Cocktail arm range from 0% absolute difference (no mortality benefit) to 20% absolute difference (that is, reduction from 25% to 5%).

4.1.2 Survivor Distribution Scenarios

For subjects in the Placebo arm that do not die, we simulate the number of days free of pressors and ventilators from the distribution shown in the left panel of Figure 8 (this is the variable denoted by Y). This distribution has a mean of 20 days.

The panel on the right then shows the induced distribution of the VVFD endpoint (this is the variable Z) for the Placebo arm. This distribution has a mean of 15 days. Note that the spike at $Z = 0$ corresponds to subjects that survive but have zero days free of pressors or ventilators, and subjects that die and thus have zero vasopressor and ventilator-free days.

Scenarios for the survivor benefit on the Virginia Cocktail arm are created by specifying a mean improvement ranging from 0 days to 1.4 days.

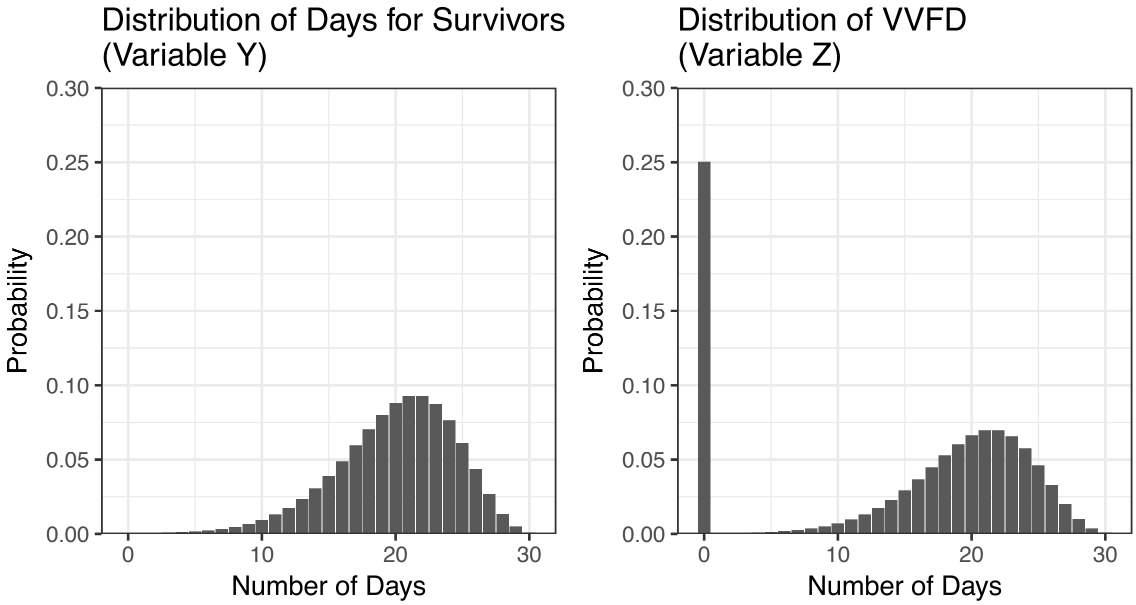

Figure 8: *Assumed distribution for vasopressor and ventilator free days in the Placebo arm*

4.1.3 Combined VVFD Scenarios

Crossing the scenarios for mortality reduction and survivor benefit creates a grid of scenarios for the VVFD endpoint. Figure 9 shows this grid along with overlaid contour lines depicting the resulting VVFD endpoint scenarios. Scenarios along each contour line have equivalent benefit on the VVFD endpoint. For example, a mortality reduction of 5% (from 25% to 20%), combined with an average 0.6 day improvement in speed of recovery for survivors, results in approximatley 1.5 days average improvement in the VVFD endpoint.

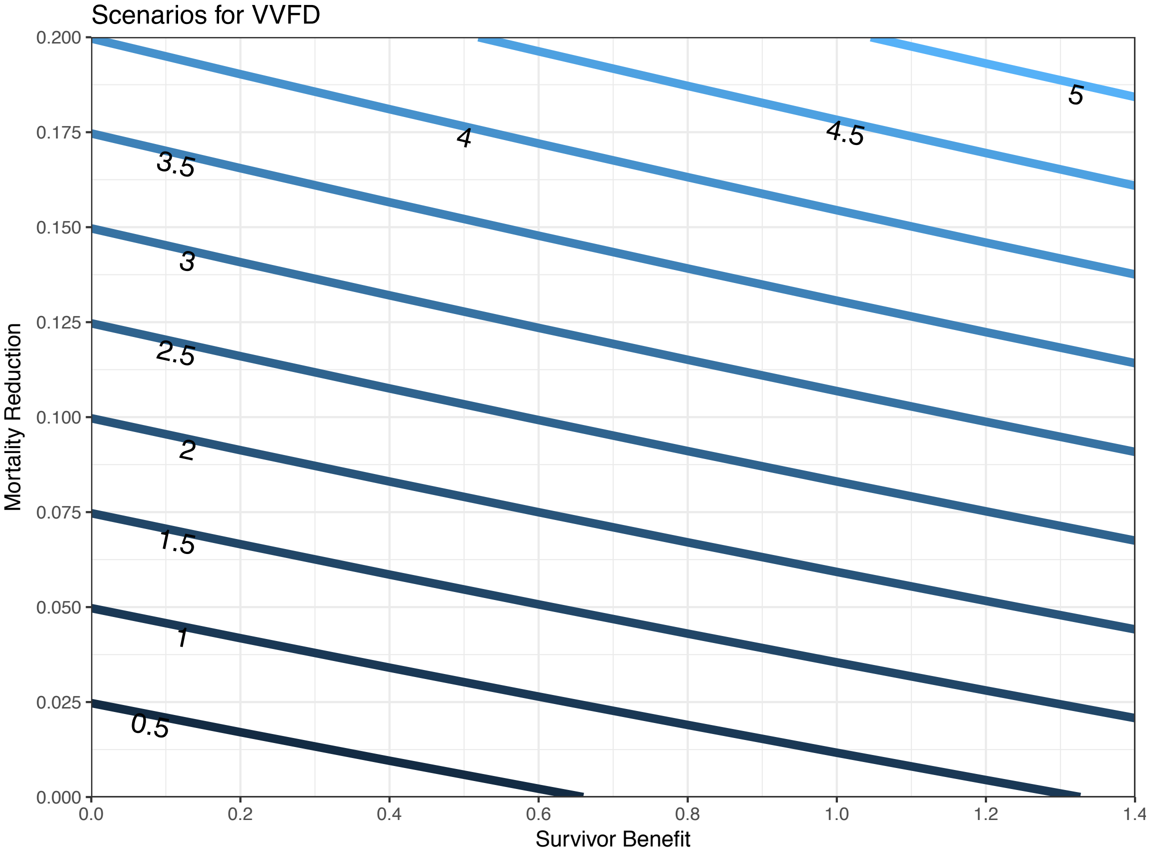

Figure 9: *Composite scenarios for VVFD*

4.2 Accrual Rate Profiles

Subject accrual to the trial is simulated from a Poisson process with an average of 80 subjects per month (so that full accrual to the maximum sample size takes on average just over 2 years). Actual accrual is simulated using exponential distributions for the intervals between subjects. Thus some simulated trials recruit more quickly than 2 years and some more slowly.

5 Operating Characteristics

For the scenarios described above, the operating characteristics of the design are demonstrated through simulation. We simulate multiple virtual trials, conduct the design as specified above, and track the behavior of each trial, including the selected sample size, the final outcome on each endpoint, etc. The results are summarized across all simulated trials for each scenario. Operating characteristics for the null scenario (no mortality benfit and no survivor benefit) are based on 10000 virtual trials. The results for all other scenarios are based on 1000 virtual trials per scenario.

5.1 Power for VVFD and Mortality

In Table 2 through Table 7, we summarize the behavior of the design, for different levels of mortality and survivor benefit, on a few key metrics:

- **Pr(trial succ.)**: the proportion of virtual trials that met the conditions for success. This includes either (1) achieving statistical significance on the mortality endpoint with a sample size of $N < 500$, or (2) achieving statistical significance on the VVFD endpoint with $N \geq 500$.
- **Pr(succ. VVFD and mortality)**: the proportion of trials that were successful both on the VVFD endpoint and on the mortality endpoint at the final analysis
- **Pr(succ. VVFD only)**: the proportion of virtual trials that met the criteria for success on VVFD at the final analysis, but not for mortality (this only applies for trials that extended the sample size to $N = 500$ or beyond, for which mortality is tested only after success on VVFD)
- **Pr(succ. mortality only)**: the proportion of virtual trials that met the criteria for success on mortality at the final analysis, but not for VVFD (this is only possible for trials with sample size $N < 500$, for which VVFD is tested only after success on mortality)
- **E[N]**: the expected sample size, averaged across all simulated trials

The total probability of success on the VVFD endpoint is then the sum of Pr(succ. VVFD and mortality) and Pr(succ. VVFD only).

Table 2 shows the probability of success when there is no true mortality benefit, and the survivor benefit ranges from 0 to 1.4 days. When there is no benefit on either component (row 1 of the table), the probability of a successful trial is 0.025 – the type I error rate. The subsequent rows of the table represent scenarios in which there is increasing benefit on the survivor effect, but no mortality effect.

Tables 3 through 7 show the operating characteristics when there is some degree of benefit on both mortality and for survivors. For example, in Table 4, when there is a 5% mortality benefit and a true 0.6-day improvement in mean free-days for survivors, the true improvement in the mean VVFD composite endpoint is 1.5 days. The probability of a successful trial is 95%. In this scenario, 76.8% of trials were successful on both VVFD and mortality. An additional 18.2% of trials were successful on VVFD but not mortality.

Table 2: *Overall operating characteristics for mortality effect = 0%*

True Mortality Effect	True Survivor Effect	True VVFD Effect	Pr(trial succ.)	Pr(succ. VVFD and mortality)	Pr(succ. VVFD only)	Pr(succ. mortality only)	E[N]
0	0.0	0.0	0.025	0.013	0.012	0	1103.8
0	0.2	0.2	0.067	0.016	0.051	0	1246.8
0	0.4	0.3	0.169	0.023	0.146	0	1409.0
0	0.6	0.5	0.365	0.025	0.340	0	1568.0
0	0.8	0.6	0.599	0.030	0.569	0	1669.0
0	1.0	0.8	0.779	0.034	0.745	0	1639.0
0	1.2	0.9	0.897	0.042	0.855	0	1548.3
0	1.4	1.1	0.960	0.031	0.929	0	1452.7

Table 3: *Overall operating characteristics for mortality effect = 2.5%*

True Mortality Effect	True Survivor Effect	True VVFD Effect	Pr(trial succ.)	Pr(succ. VVFD and mortality)	Pr(succ. VVFD only)	Pr(succ. mortality only)	E[N]
0.025	0.0	0.5	0.154	0.127	0.026	0.001	1365.2
0.025	0.2	0.7	0.315	0.187	0.128	0.000	1536.7
0.025	0.4	0.8	0.532	0.252	0.280	0.000	1654.4
0.025	0.6	1.0	0.708	0.266	0.442	0.000	1720.8
0.025	0.8	1.1	0.873	0.270	0.603	0.000	1703.9
0.025	1.0	1.3	0.951	0.270	0.681	0.000	1660.8
0.025	1.2	1.4	0.982	0.259	0.723	0.000	1601.3
0.025	1.4	1.6	0.996	0.254	0.742	0.000	1523.9

Table 4: *Overall operating characteristics for mortality effect = 5%*

True Mortality Effect	True Survivor Effect	True VVFD Effect	Pr(trial succ.)	Pr(succ. VVFD and mortality)	Pr(succ. VVFD only)	Pr(succ. mortality only)	E[N]
0.05	0.0	1.0	0.447	0.427	0.020	0.000	1512.5
0.05	0.2	1.2	0.654	0.605	0.048	0.001	1522.0
0.05	0.4	1.3	0.816	0.683	0.130	0.003	1504.6
0.05	0.6	1.5	0.950	0.768	0.182	0.000	1501.2
0.05	0.8	1.6	0.972	0.759	0.213	0.000	1473.0
0.05	1.0	1.8	0.976	0.731	0.245	0.000	1428.8
0.05	1.2	2.0	0.992	0.756	0.236	0.000	1421.2
0.05	1.4	2.1	0.994	0.748	0.246	0.000	1427.0

***Table 5:** Overall operating characteristics for mortality effect = 7.5%*

True Mortality Effect	True Survivor Effect	True VVFD Effect	Pr(trial succ.)	Pr(succ. VVFD and mortality)	Pr(succ. VVFD only)	Pr(succ. mortality only)	E[N]
0.075	0.0	1.5	0.796	0.783	0.002	0.011	1381.8
0.075	0.2	1.7	0.900	0.891	0.006	0.003	1258.5
0.075	0.4	1.8	0.954	0.936	0.012	0.006	1129.6
0.075	0.6	2.0	0.972	0.956	0.015	0.001	1085.1
0.075	0.8	2.2	0.979	0.962	0.017	0.000	1056.4
0.075	1.0	2.3	0.985	0.961	0.023	0.001	1098.0
0.075	1.2	2.5	0.988	0.969	0.018	0.001	1050.6
0.075	1.4	2.7	0.981	0.959	0.022	0.000	1041.2

***Table 6:** Overall operating characteristics for mortality effect = 10%*

True Mortality Effect	True Survivor Effect	True VVFD Effect	Pr(trial succ.)	Pr(succ. VVFD and mortality)	Pr(succ. VVFD only)	Pr(succ. mortality only)	E[N]
0.1	0.0	2.0	0.934	0.903	0.000	0.031	1039.2
0.1	0.2	2.2	0.958	0.938	0.000	0.020	952.6
0.1	0.4	2.3	0.966	0.954	0.001	0.011	845.5
0.1	0.6	2.5	0.969	0.960	0.002	0.007	813.3
0.1	0.8	2.7	0.967	0.963	0.002	0.002	761.0
0.1	1.0	2.9	0.960	0.959	0.001	0.000	767.6
0.1	1.2	3.0	0.985	0.983	0.002	0.000	751.2
0.1	1.4	3.2	0.980	0.978	0.002	0.000	741.7

Table 7 reports the operating characteristics for a very large mortality benefit of 20%. The power of the trial is near 100%, with most trials meeting success on both endpoints. Additionally, the expected sample size under this set of scenarios is quite small, as most trials stop at one of the early interims under these conditions.

Table 7: *Overall operating characteristics for mortality effect = 20%*

True Mortality Effect	True Survivor Effect	True VVFD Effect	Pr(trial succ.)	Pr(succ. VVFD and mortality)	Pr(succ. VVFD only)	Pr(succ. mortality only)	E[N]
0.2	0.0	4.0	0.990	0.833	0	0.157	269.8
0.2	0.2	4.2	0.994	0.886	0	0.108	270.4
0.2	0.4	4.4	0.990	0.911	0	0.079	264.4
0.2	0.6	4.6	0.995	0.959	0	0.036	267.8
0.2	0.8	4.8	0.994	0.981	0	0.013	266.9
0.2	1.0	5.0	0.992	0.984	0	0.008	270.3
0.2	1.2	5.1	0.993	0.989	0	0.004	269.6
0.2	1.4	5.3	0.987	0.987	0	0.000	266.8

Figure 10 visually displays the probability of trial success over the grid of scenarios (for 10% and smaller mortality benefit). In the bottom left corner, the probability of success under the null scenario is 0.025, corresponding to the type I error rate of the design with the futility rule enabled. Moving from bottom left to top right on the grid corresponds to increasing benefit. For example, for true 5% improvement on mortality and 0.6 days mean improvement for survivors (true VVFD benefit of 1.5 average days), the power of the trial is 0.95, as reported in Table 4.

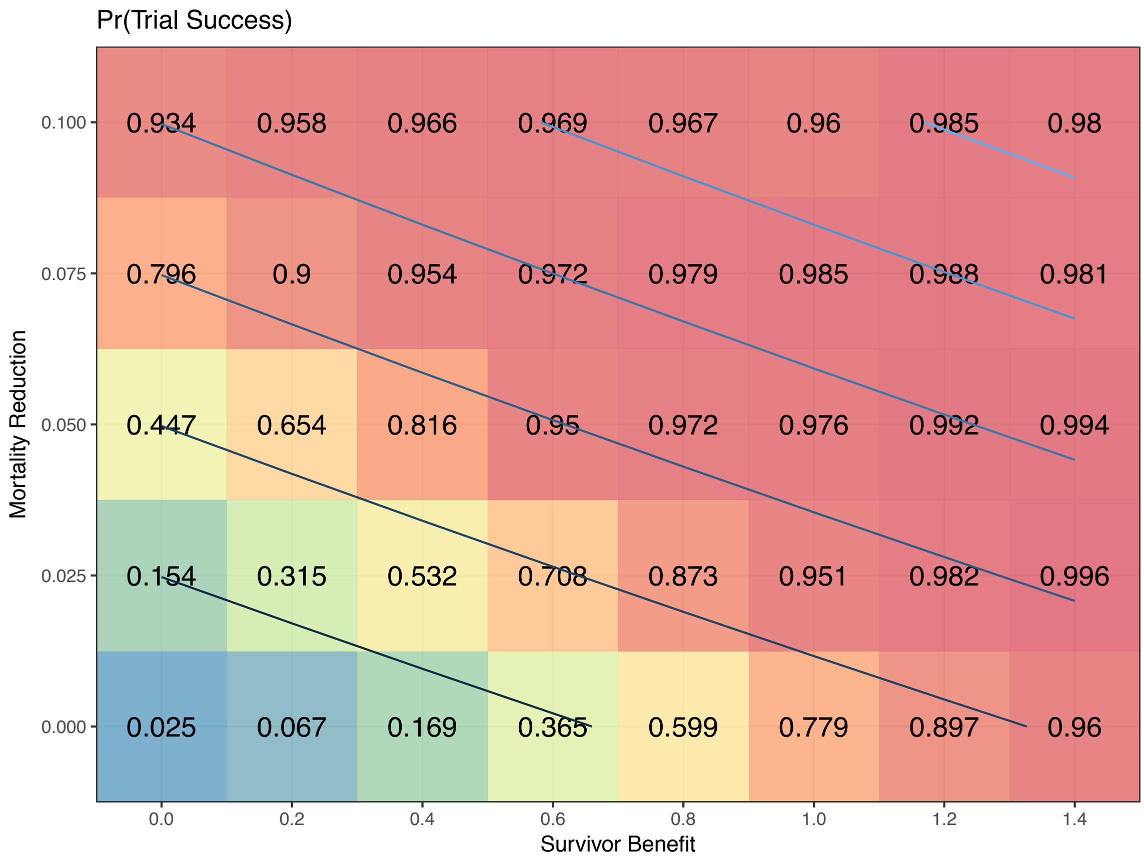

Figure 10: *Probability of trial success*

Similarly, Figure 11 shows the probability of winning both endpoints. For a 5% mortality benefit, the probability of winning both endpoints is below 80%, reflecting the lower power for mortality than for VVFD.

In the null scenario, across all simulated trials, and 1.3% of trials were successful on both endpoints.

When the true mortality benefit is a 5% reduction, and the survivor benefit is 0.6 days on average results in the truth on the composite endpoint being a 1.5-day mean improvement in VVFD. Under this scenario, 76.8% of trials were successful on the both endpoints.

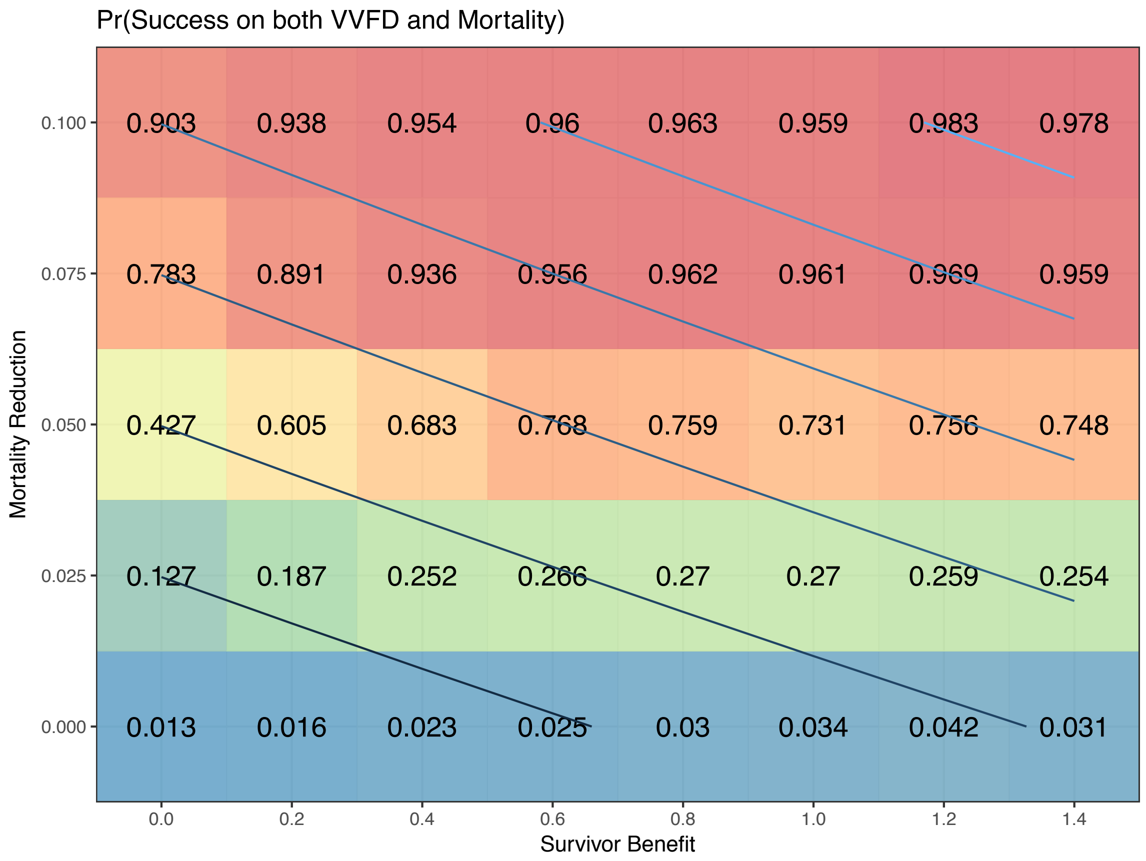

Figure 11: *Probability of success for VVFD and mortality*

5.2 Summary of Sample Size Selection

Figure 12 illustrates the average sample size across trials for the grid of scenarios (for 10% and smaller mortality benefit). Scenarios that result in the smallest sample sizes are shaded dark green, while dark purple corresponds to higher average sample sizes. The smallest sample sizes occur for the scenarios with 20% mortality reduction (not shown in the Figure). From Table 7, the average sample size in those scenarios is less than 300 subjects, reflecting high probability of stopping accrual at an early interim based on the large mortality benefit. Scenarios with large improvements in both components of the endpoint (top right corner of the Figure) tend to have the next smallest sample sizes (< 800), as these trials often stop early for success on both endpoints. Scenarios with slight to moderate effects on mortality tend to result in larger trials, due to the bypass rule. When the mortality benefit is quite large (e.g. 10%), bypass is rarely necessary, so sample sizes tend to be smaller.

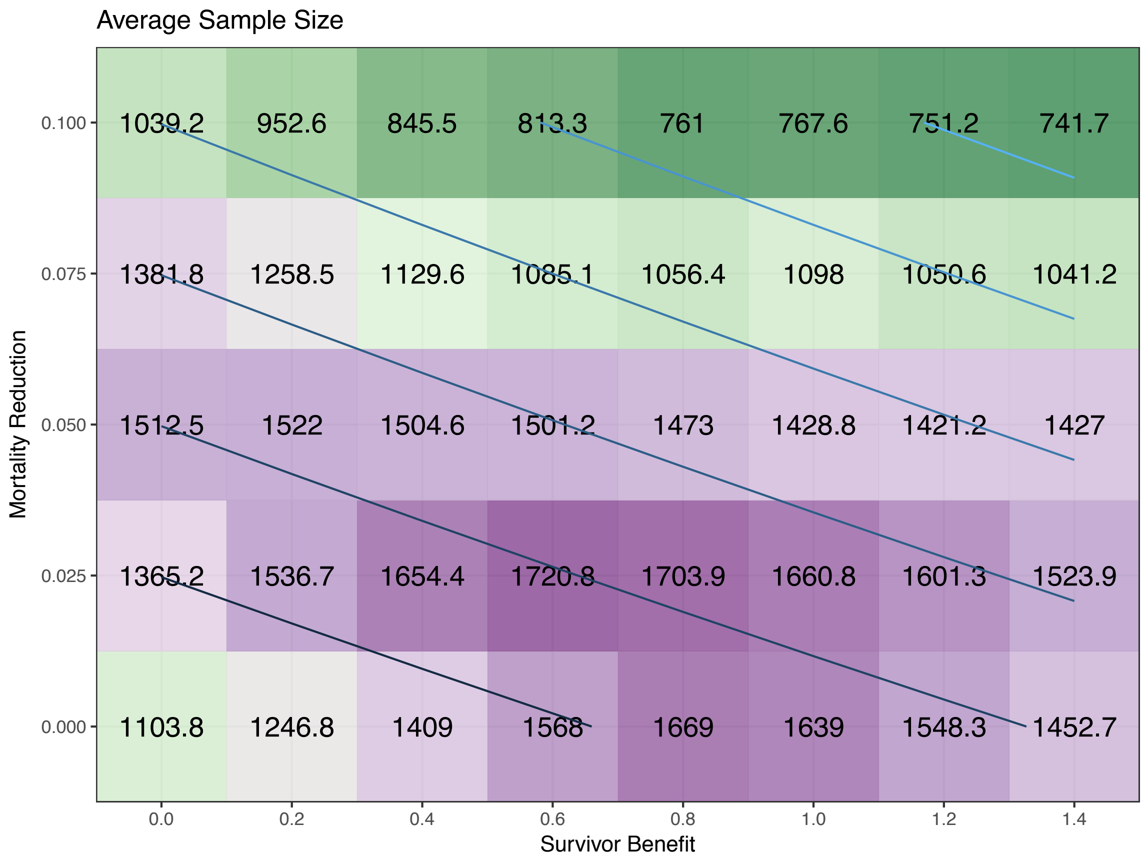

Figure 12: *Average Sample Size*

For a few select scenarios, we summarize the selected sample size and reason for stopping accrual. Table 8 illustrates the null scenario, with each row representing a different reason for stopping accrual, and each column a selected sample size. Under the null, 84.6% of trials stop for futility. About 29.6% of trials end in futility at the *N* = 500 interim, with an additional 33.8% stopping for futility at the *N* = 1000 interim, and 21.2% at the *N* = 1500 interim. About 14.6% of trials enrolled to the maximum sample size.

Table 8: *Summary of Sample Size Selection for Mortality Effect = 0%, Survivor Effect = 0 days*

	N = 200	N = 300	N = 400	N = 500	N = 1000	N = 1500	N = 2000	Total
Futility	--	--	--	0.296	0.338	0.212	--	0.846
Expected success (mortality)	0.001	0	0	--	--	--	--	0.001
Expected success (both endpoints)	--	--	--	0.001	0.003	0.003	--	0.007
Expected success (VVFD only)	--	--	--	0.000	0.000	0.000	--	0.000
Maximum Sample Size	--	--	--	--	--	--	0.146	0.146
Total	0.001	0	0	0.297	0.341	0.215	0.146	1.000

For the scenario with true 5% reduction in mortality rate and 0.6-day survivor benefit, about 45% of trials enrolled to the maximum sample size (Table 9). Approximately 52.1% of trials stopped accrual early for expected success (2.2% stopped at an early interim for expected mortality success, 47.7% of the time, the trial stopped for expected on both endpoints, while 2.2% of trials stopped for expected success on only VVFD).

Table 9: *Summary of Sample Size Selection for Mortality Effect = 5%, Survivor Effect = 0.6 days*

	N = 200	N = 300	N = 400	N = 500	N = 1000	N = 1500	N = 2000	Total
Futility	--	--	--	0.016	0.006	0.010	--	0.032
Expected success (mortality)	0.007	0.007	0.008	--	--	--	--	0.022
Expected success (both endpoints)	--	--	--	0.080	0.190	0.207	--	0.477
Expected success (VVFD only)	--	--	--	0.000	0.004	0.018	--	0.022
Maximum Sample Size	--	--	--	--	--	--	0.447	0.447
Total	0.007	0.007	0.008	0.096	0.200	0.235	0.447	1.000

When there is in truth a 20% improvement in mortality (Table 10), most trials stop accrual at or before $N = 500$ subjects. Almost 50% of trials stopped for expected mortality success at the first interim. None of the trials enrolled past $N = 1000$ in the simulations.

Table 10: *Summary of Sample Size Selection for Mortality Effect = 20%, Survivor Effect = 0.6 days*

	N = 200	N = 300	N = 400	N = 500	N = 1000	N = 1500	N = 2000	Total
Futility	--	--	--	0.000	0.000	0	--	0.000
Expected success (mortality)	0.497	0.362	0.112	--	--	--	--	0.971
Expected success (both endpoints)	--	--	--	0.028	0.001	0	--	0.029
Expected success (VVFD only)	--	--	--	0.000	0.000	0	--	0.000
Maximum Sample Size	--	--	--	--	--	--	0	0.000
Total	0.497	0.362	0.112	0.028	0.001	0	0	1.000

5.3 Summary of Bypass

An important feature of this trial is the ability to bypass an early stop on VVFD in the hope of picking up the additional endpoint of mortality (note that the bypass rules only apply to the interims at $N = 500$ and beyond. Table 11 summarizes how often the design bypasses when the true mortality reduction is 0% and the true survivor benefit is 0 days (null scenario). Each column represents the additional sample size that was added after bypass. For example, +0 indicates that no bypass was taken, while +500 indicates that bypass was taken, but the trial stopped at the next planned analysis, resulting in an increase of 500 additional subjects. For each possible increase, we summarize the ultimate outcome of the trial.

Table 11: *Summary of Bypass for Mortality Effect = 0%, Survivor Effect = 0 days*

	+0	+500	+1000	+1500	Total
Succ. both endpoints	0.010	0.002	0.001	0.000	0.013
Succ. VVFD only	0.008	0.001	0.001	0.001	0.012
Succ. mort only	0.000	0.000	0.000	0.000	0.000
Fail both endpoints	0.968	0.001	0.003	0.003	0.975
Total	0.987	0.004	0.005	0.004	1.000

Table 12: *Summary of Bypass for Mortality Effect = 5%, Survivor Effect = 0.6 days*

	+0	+500	+1000	+1500	Total
Succ. both endpoints	0.385	0.229	0.125	0.029	0.768
Succ. VVFD only	0.079	0.051	0.037	0.015	0.182
Succ. mort only	0.000	0.000	0.000	0.000	0.000
Fail both endpoints	0.049	0.000	0.000	0.001	0.050
Total	0.513	0.280	0.162	0.045	1.000

In the null scenario, 98.7% of trials never bypass.

Table 12 summarizes bypass for the scenario when the true mortality reduction is 5% and the true survivor benefit is 0.6 days. No bypass was taken in 51% of trials, and most of those trials (38.5% out of 1000 trials) were successful on both endpoints. The trial took a bypass in 48.7% of trials, most often adding 500 additional subjects before stopping accrual. Out of 1000 trials, 280 trials added 500 additional subjects, and 229 of those trials ended with a successful outcome on both endpoints.

6 Computational Details

The simulations were run using R (R Core Team 2016) version 3.3.0. Bayesian computations were performed using JAGS (Just Another Gibbs Sampler), specifically

the rjags R package, version 4-6. The predictive probability calculations were based on Markov chain Monte Carlo (MCMC) chains of length 2500 after a burn-in of 2500.

7 APPENDIX: Statistical Model for Adaptive Design Decisions

At each interim analysis, there will be subjects whose final outcome is unknown (e.g. subjects who have been enrolled but whose data is not yet available, or subjects not yet enrolled). To make decisions regarding sample size selection, Bayesian predictive distributions are employed for the multiple imputation of outcomes for such subjects. The predictive probabilities are computed using the following model.

Let X_{ij} be the mortality outcome ($X = 1$ for death, $X = 0$ for survival) for subject i assigned to arm j ($j = PBO$ for p=Placebo, $j = VC$ for the Virginia Cocktail). Let Y_{ij} be the number of days free of pressors and ventilators, conditional on survival. Then the primary endpoint, Z_{ij} is constructed as:

$$Z_{ij} = \begin{cases} 0 & \text{if } X_{ij} = 1; \\ Y_{ij} & \text{if } X_{ij} = 0. \end{cases}$$

We model the components of the VVFD endpoint separately. Let q_j be the mortality rate on arm j. We transform the mortality rates onto the log-odds scale so that:

$$\log\left(\frac{q_{PBO}}{1 - q_{PBO}}\right) = \gamma$$

for the Placebo arm and

$$\log\left(\frac{q_{VC}}{1 - q_{VC}}\right) = \gamma + \delta$$

for the Virginia Cocktail arm. Thus the parameter δ represents the mortality benefit of the Virginia Cocktail, relative to Placebo, on the log-odds scale.

The log-odds rate on the Placebo arm is modeled as

$$\gamma \sim N(0, 2^2),$$

which is approximately uniform when transformed back to the probability scale. The mortality effect has non-informative prior distribution

$$\delta \sim N(0, 10^2).$$

The model for survivor benefit is as follows: For the Placebo arm, the probability that a subject who survives has k days free of pressors and ventilators is

$$Pr(Y = k | X = 0, j = PBO) = \pi_k$$

for $k = 0, \ldots, 30$, subject to

$$\sum \pi_k = 1.$$

For a subject on the Virginia Cocktail arm, we model the effect on survivors using an exponential family whose sufficient statistic is the number of VVFD:

$$Pr(Y = k | X = 0, j = VC) = c\pi_k e^{k\theta},$$

where c is a normalization constant. Thus the treatment effect for the primary endpoint of VVFD has two components: the effect on mortality, δ, and the effect on the survivors, θ. Note that subjects who die are modeled separately from subjects who survive, but nevertheless accumulate zero VVFD. These subjects are handled in the same way in the final analysis (Wilcoxon test).

The prior distribution for the Placebo arm is specified as

$$(\pi_1, \ldots, \pi_{30}) \sim \text{Dirichlet}\left(\frac{1}{3}, \ldots, \frac{1}{3}\right),$$

which has approximately 10 observations worth of prior weight. For the survivor benefit, we use

$$\theta \sim N(0, 1^2).$$

Based on this model, we compute two predictive probabilities for the P&VFD endpoint for use in interim decisions:

- PP_{VVFD} (current N): the predictive probability of success on the VVFD endpoint (with $\alpha = 0.022$) if enrollment stops with the current sample size, and all currently enrolled subjects are followed to their primary endpoint.
- PP_{VVFD} (max N): the predictive probability of success on the primary VVFD endpoint (with $\alpha = 0.022$) if enrollment continues to the maximum number of subjects ($N = 2000$).

Similarly, we compute two predictive probabilites for the mortality endpoint. For these computations, we rely on a simpler Beta-Binomial model, with independent non-informative Beta(0.5, 0.5) priors on each q_j.

- PP_{mort} (current N): the predictive probability of success on the mortality endpoint (with $\alpha = 0.001$ for $N < 500$ and $\alpha = 0.024$ for $N \geq 500$) if enrollment stops with the current sample size, and all currently enrolled subjects are followed to their primary endpoint.
- PP_{mort} (max N): the predictive probability of success on the mortality endpoint (with $\alpha = 0.024$) if enrollment continues to the maximum number of subjects ($N = 2000$).

These predictive probabilities will be used for sample size selection decisions.
